# RNF220-mediated K63-linked polyubiquitination stabilizes Olig proteins during oligodendroglial development and myelination

**DOI:** 10.1126/sciadv.adk3931

**Published:** 2024-02-07

**Authors:** Yuwei Li, Li Pear Wan, Ning-Ning Song, Yu-Qiang Ding, Shuhua Zhao, Jianqin Niu, Bingyu Mao, Nengyin Sheng, Pengcheng Ma

**Affiliations:** ^1^ State Key Laboratory of Genetic Resources and Evolution, Kunming Institute of Zoology, Chinese Academy of Sciences, Kunming 650223, China.; ^2^ Kunming College of Life Science, University of Chinese Academy of Sciences, Kunming 650223, China.; ^3^ Key Laboratory of Animal Models and Human Disease Mechanisms of Yunnan Province, Kunming Institute of Zoology, Chinese Academy of Sciences, Kunming, Yunnan 650223, China.; ^4^ Department of Laboratory Animal Science, Fudan University, Shanghai 200032, China.; ^5^ State Key Laboratory of Medical Neurobiology and MOE Frontiers Center for Brain Science, Institutes of Brain Science, Fudan University, Shanghai 200032, China.; ^6^ First Affiliated Hospital of Kunming Medical University, Kunming 650032, China.; ^7^ Department of Histology and Embryology, Third Military Medical University, Chongqing 400038, China.; ^8^ Center for Excellence in Animal Evolution and Genetics, Chinese Academy of Sciences, Kunming 650223, China.

## Abstract

Maldevelopment of oligodendroglia underlies neural developmental disorders such as leukodystrophy. Precise regulation of the activity of specific transcription factors (TFs) by various posttranslational modifications (PTMs) is required to ensure proper oligodendroglial development and myelination. However, the role of ubiquitination of these TFs during oligodendroglial development is yet unexplored. Here, we find that RNF220, a known leukodystrophy-related E3 ubiquitin ligase, is required for oligodendroglial development. RNF220 depletion in oligodendrocyte lineage cells impedes oligodendrocyte progenitor cell proliferation, differentiation, and (re)myelination, which consequently leads to learning and memory defects. Mechanistically, RNF220 targets Olig1/2 for K63-linked polyubiquitination and stabilization during oligodendroglial development. Furthermore, in a knock-in mouse model of leukodystrophy-related RNF220^R365Q^ mutation, the ubiquitination and stabilization of Olig proteins are deregulated in oligodendroglial cells. This results in pathomimetic oligodendroglial developmental defects, impaired myelination, and abnormal behaviors. Together, our evidence provides an alternative insight into PTMs of oligodendroglial TFs and how this essential process may be implicated in the etiology of leukodystrophy.

## Teaser

RNF220 regulates oligodendroglial development and myelination by modulating Olig1/2 polyubiquitination and stabilization.

## INTRODUCTION

Myelination by mature oligodendrocytes (OLs) enables saltatory conduction of action potentials and provides long-term trophic support for neuronal axons, maintaining neural integrity throughout the central nervous system (CNS) (*1*). During development, neural stem cells–derived OL progenitor cells (OPCs) proliferate to fill up the CNS, differentiate into OLs, and terminally wrap around axons for proper myelination (*2*). During these processes, defects in oligodendroglial development and myelination can cause various neurological disorders, such as leukodystrophy, which is also a rare genetic disorder (*3*). Patients with leukodystrophy can feature cognitive symptoms, intellectual disabilities, or social dysfunction (*4**–**6*). However, knowledge about the etiological mechanism of leukodystrophy is lacking, especially with regards to the regulatory factors needed for oligodendroglial development.

Precise and dynamic regulation of transcription factor (TF) activities is critical for proper oligodendroglial development (*7*). Posttranslational modifications (PTMs) are involved in modulating TF subcellular localization and also the protein interacting complexes. Through this, PTMs are able to regulate specific processes of oligodendroglial development and regeneration (*7*). For instance, it has been reported that acetylation and phosphorylation are required for Olig1 translocation from the nucleus to the cytoplasm during oligodendroglial differentiation (*8*, *9*); and dephosphorylation of Ser^147^ primes Olig2 for the function of OPC specification via interacting specific cofactors (*10*). It is known that E3 ubiquitin ligase–mediated protein homeostasis is crucial for neural development by influencing the protein stabilities of various master TFs that govern neurogenesis such as Pax6 and Ascl1 (*11*, *12*), as well as astrogliogenesis like Ets variant transcriptional factors (ETVs) (*13*). Hence, activities of E3 ubiquitin ligases should be precisely regulated during neural developmental processes. However, it remains elusive whether and how regulatory TFs are modulated by ubiquitination during oligodendroglial development.

Among the estimated 600 to 700 human E3 ubiquitin ligase–encoded genes, 83 genes are mutated and implicated in 70 different types of neurological diseases (*14*). From these diseases, one typical neurodevelopmental disorder is hypomyelinating leukodystrophy (HLD) which is characterized by a primary lack of myelin deposition (*3*). Among the 26 types of established HLD recorded in the Online Mendelian Inheritance in Man (OMIM) database, HLD-23 is caused by mutations of the gene *RNF220* (*RING finger protein 220*) (*15*), which encodes a RING-type E3 ubiquitin ligase. Individuals harboring homozygous R363Q or R365Q mutation of RNF220 have symptoms of leukodystrophy and corpus callosum agenesis, as well as intellectual disability (*16*, *17*). However, the physiological roles of RNF220 during oligodendroglial development and its ability to regulate TFs during leukodystrophy onset are unclear.

In this study, we found that the E3 ubiquitin ligase RNF220 is required for oligodendroglial development, and its specific depletion in OL lineage cells impairs OPC proliferation, differentiation, subsequent myelination, as well as remyelination after demyelinating injury in the adult mouse brains. Furthermore, we showed that Olig1 and Olig2 are two direct ubiquitination substrates of RNF220, and the protein stabilities of these two TFs are maintained by RNF220 through K63-linked polyubiquitination. We were able to replicate leukodystrophy-like symptoms in a pathomimetic RNF220^R365Q^ knock-in mouse model, which represents deregulated K63 ubiquitination of Olig proteins and impediments of oligodendroglial differentiation and myelination.

## RESULTS

### RNF220 is required for oligodendroglial development and myelination

To examine the expression of RNF220 in OL lineage cells, OPCs and differentiated OLs were purified with either platelet-derived growth factor receptor α–positive (PDGFRα^+^) or oligodendrocyte cell surface antigen 4 (O4^+^) beads, by using astrocyte cell surface antigen-2 (ASCA2^+^) beads purification of astrocytes as a control (fig. S1A). Quantitative polymerase chain reaction (PCR) (fig. S1B) and Western blot (WB) (fig. S1C) results indicated that the expression levels of RNF220 mRNA and protein were relatively constant in OPC and OL cells. Moreover, immunofluorescence staining on corpus callosum sections showed that RNF220 was mainly observed in the cytoplasm of PDGFRα^+^ OPCs (fig. S1D) and CC1^+^ OLs (fig. S1E) while the intensity was much lower in the nucleus.

To analyze the function of RNF220 during oligodendroglial development, we depleted *RNF220* in oligodendroglial lineage cells by crossing conditional *RNF220^flox/flox^* mice (*18*) with *Olig1-Cre* mice, which is a pan-OL lineage Cre mouse line (*19*), and the resultant three mouse lines, namely, *RNF220^wt/wt^;Olig1-Cre^+/−^* (hereafter referred to as *RNF220-WT*), *RNF220^flox/wt^;Olig1-Cre^+/−^* (*RNF220-cHet*), and *RNF220^flox/flox^;Olig1-Cre^+/−^* (*RNF220-cKO*), were used for subsequent studies. The deletion of RNF220 in these mice was confirmed through checking relative mRNA and protein levels (fig. S2, A and B). Immunofluorescence staining analyses further showed that RNF220 expression in Sox10^+^ OL lineage cells of corpus callosum on postnatal day 21 (P21) was also reduced (fig. S2C). Moreover, all the three lines of both sexes were born at Mendelian ratios and there was no significant difference of survival rate, general appearance, body weight, or brain weight at adult age (fig. S2, D to F).

**Fig. 1.**
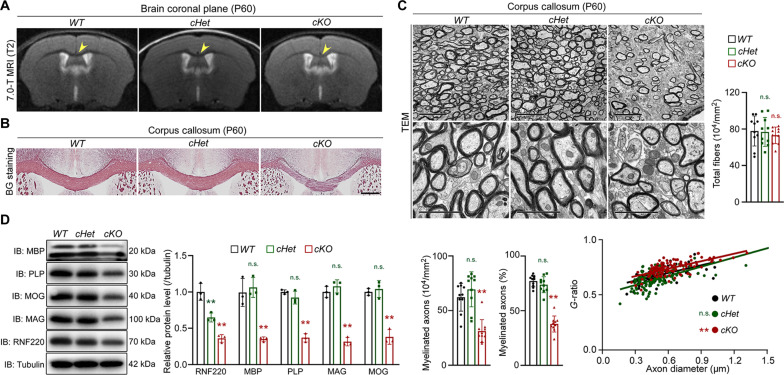
RNF220 knockout leads to myelination defects in mice. (**A**) Representative T2-weighted mouse brain MRI scanning of P60 *RNF220-WT* (*n* = 3), *RNF220-cHet* (*n* = 3), and *RNF220-cKO* (*n* = 3) mice. The arrows indicate the corpus callosum. (**B**) Representative images showing myelin staining (BG) in corpus callosum regions of P60 *RNF220-WT* (*n* = 3), *RNF220-cHet* (*n* = 3), and *RNF220-cKO* (*n* = 3) mice. (**C**) Electron microscopy images of the corpus callosum transverse sections from P60 *RNF220-WT* (*n* = 8), *RNF220-cHet* (*n* = 8), and *RNF220-cKO* (*n* = 8) mice with indicated magnifications. Scale bars, 5 μm for upper panels and 2 μm for lower panels. Bar graphs (mean ± SD) show quantification of the total fiber number and the number or percentage of myelinated axons, and scatterplots show *g*-ratios relative to axon diameter. (**D**) WB analyses of protein expression of RNF220, MBP, PLP, MAG, and MOG in the cerebral cortex of P21 *RNF220-WT* (*n* = 3), *RNF220-cHet* (*n* = 3), and *RNF220-cKO* (*n* = 3) mice. α-Tubulin was used as the internal controls. Bar graphs (mean ± SD) show the relative levels normalized against indicated proteins expression in the respective wild-type (WT) controls. IB, immunoblotting. Statistical analyses are compared to respective control with Mann-Whitney *U* test with Bonferroni correction. n.s. (not significant), *P* > 0.05; **P < 0.01.

As RNF220 mutation is regarded as a pathological cause of leukodystrophy, T2-weight magnetic resonance imaging (MRI) analysis was used to examine white matter volume of corpus callosum in the above three mouse lines. The results showed that the signal intensity was specifically and significantly increased in adult *RNF220-cKO* mice as compared to *RNF220-WT* and *RNF220-cHet*, suggesting that RNF220 deficiency may lead to myelination defects in mouse brains (Fig. 1A). Furthermore, black-gold (BG) staining was further used to examine the axonal fibers of corpus callosum in these mice, where consistently severe hypomyelination was only found in *RNF220-cKO* mice (Fig. 1B). When transmission electron microscopy (TEM) imaging was applied to analyze the myelin ultrastructure, although the number of total axons in the corpus callosum was not changed (*WT*: 77.85 ± 16.39 × 10^4^/mm^2^; *cHet*: 76.80 ± 15.86 × 10^4^/mm^2^; *cKO*: 73.11 ± 9.05 × 10^4^/mm^2^; Fig. 1C), the number (*WT*: 62.34 ± 12.77 × 10^4^/mm^2^; *cHet*: 69.58 ± 16.04 × 10^4^/mm^2^; *cKO*: 31.24 ± 10.56 × 10^4^/mm^2^; Fig. 1C) and percentage (*WT*: 77.20 ± 5.20%; *cHet*: 73.80 ± 7.33%; *cKO*: 37.90 ± 7.33%; Fig. 1C) of myelinated axons, and myelin thickness (*g*-ratios: *WT*: 0.68 ± 0.07; *cHet*: 0.65 ± 0.09; *cKO*: 0.74 ± 0.06; Fig. 1C) were specifically and markedly reduced in the corpus callosum of adult *RNF220-cKO* mice. Accordingly, both the mRNA (fig. S2G) and protein levels (RNF220: *WT*: 1.00 ± 0.12, *cHet*: 0.65 ± 0.06, *cKO*: 0.36 ± 0.05; myelin basic protein (MBP): *WT*: 1.00 ± 0.19, *cHet*: 1.06 ± 0.14, *cKO*: 0.35 ± 0.03; proteolipid protein (PLP): *WT*: 1.00 ± 0.03, *cHet*: 0.92 ± 0.09, *cKO*: 0.37 ± 0.06; myelin-associated glycoprotein (MAG): *WT*: 1.00 ± 0.08, *cHet*: 1.08 ± 0.09, *cKO*: 0.32 ± 0.06; myelin oligodendrocyte glycoprotein (MOG): *WT*: 1.00 ± 0.05, *cHet*: 1.04 ± 0.12, *cKO*: 0.38 ± 0.10; Fig. 1D) of myelin-related molecules, including MAG, MOG, PLP, and MBP, were significantly decreased in *RNF220-cKO* adult brains. In addition, the myelin ultrastructure in the spinal cord in these mice was also analyzed by TEM imaging analyses, and similar results were obtained to show specific impairment of the axon myelination in adult *RNF220-cKO* mice (fig. S3). Together, these results indicate that RNF220 is required for myelination in mice.

**Fig. 2.**
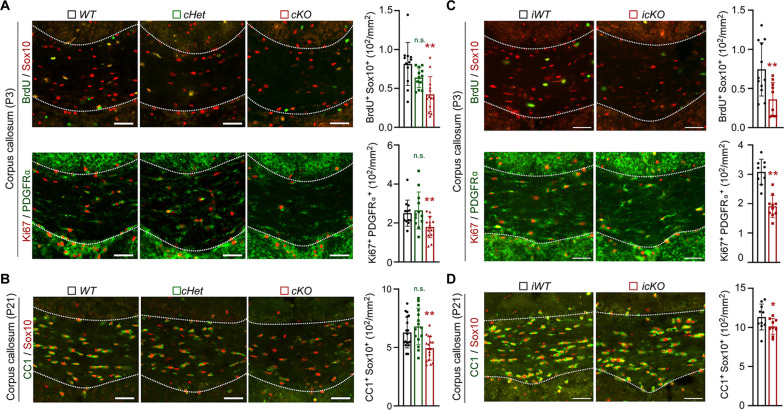
RNF220 knockout impairs oligodendroglial development defects in mice. (**A**) Immunofluorescence staining assays of BrdU and Sox10, or Ki67 and PDGFRα in the corpus callosum regions of *RNF220-WT* (*n* = 3), *RNF220-cHet* (*n* = 3), and *RNF220-cKO* (*n* = 3) mice on P3. Bar graphs (mean ± SD) show the quantification of BrdU^+^Sox10^+^ and Ki67^+^PDGFRα^+^ cells. (**B**) Immunofluorescence staining assays of CC1 and Sox10 in the corpus callosum regions of *RNF220-WT* (*n* = 3), *RNF220-cHet* (*n* = 3), and *RNF220-cKO* (*n* = 3) mice on P21. Bar graphs (mean ± SD) show the quantification of CC1^+^Sox10^+^ cells. (**C**) Immunofluorescence costaining of BrdU and Sox10, or Ki67 and PDGRFα in the corpus callosum of *RNF220-iWT* (*n* = 3) and *RNF220-icKO* (*n* = 3) on P3, and bar graphs (mean ± SD) show the quantification of BrdU^+^Sox10^+^ cells, and Ki67^+^ PDGRFα^+^ cells. (**D**) Immunofluorescence staining of CC1 and Sox10 in the corpus callosum of *RNF220-iWT* (*n* = 3) and *RNF220-icKO* (*n* = 3) on P21, and bar graphs (mean ± SD) show the quantification of CC1^+^Sox10^+^ cells. Scale bars, 50 μm. Statistical analyses are compared to respective control with Mann-Whitney *U* test with Bonferroni correction. n.s. (not significant), *P* > 0.05; *P < 0.05; **P < 0.01.

As RNF220 is highly expressed in OL lineage cells (fig. S1), we wondered whether the corpus callosum agenesis in *RNF220-cKO* mice can be attributed to the maldevelopment of oligodendroglia cells. To this end, we first examined OPC proliferation during development and found that both 5-bromo-2′-deoxyuridine (BrdU) labeling and Ki67 staining in the corpus callosum of P3 mice were significantly reduced after RNF220 knockout (BrdU^+^Sox10^+^ cells: *WT*: 0.81 ± 0.28 × 10^2^/mm^2^; *cHet*: 0.64 ± 0.13 × 10^2^/mm^2^; *cKO*: 0.42 ± 0.23 × 10^2^/mm^2^; Ki67^+^PDGFRα^+^ cells: *WT*: 2.51 ± 0.67 × 10^2^/mm^2^; *cHet*: 2.64 ± 0.95 × 10^2^/mm^2^; *cKO*: 1.80 ± 0.55 × 10^2^/mm^2^; Fig. 2A). Correspondingly, RNF220 knockdown in MOPC cells (fig. S4A), which is a mouse OPC cell line, also exhibited a decreased proliferating rate by analyzing 5-ethynyl-2-deoxyuridine (EdU) incorporation (fig. S4B) and the expression of OPC markers (fig. S4C). Coimmunostaining of the OPC marker Sox10 and the OL marker CC1 was used to analyze OL differentiation, and we further found that the number of CC1^+^Sox10^+^ cells was significantly less in the corpus callosum of P21 *RNF220-cKO* mice (*WT*: 6.28 ± 1.32 × 10^2^/mm^2^; *cHet*: 6.80 ± 1.57 × 10^2^/mm^2^; *cKO*: 4.94 ± 1.02 × 10^2^/mm^2^; Fig. 2B). In addition, when MOPC cells were induced to differentiate using T3 supplements (fig. S4D), the expression levels of all the examined myelin-related genes were down-regulated after RNF220 was knocked down while were restored by small interfering RNA (siRNA)–resistant RNF220 coexpression (fig. S4E). It is known that there is a chronological procedure of olilgodendrolial development in postnatal mouse brains: before P7 OPCs are mainly proliferated and after that OL cells are differentiated (*20*). Therefore, to examine whether the impaired OL differentiation after RNF220 depletion is a secondary effect to the early deregulated proliferation, *RNF220^flox/flox^* mice were crossed with the *PDGFR*α*-CreER* transgenic mouse line, an OPC-specific tamoxifen-inducible Cre line (*21*), to obtain the three mouse lines (*RNF220^wt/wt^;PDGFR*α*-CreER, RNF220-iWT*; *RNF220^flox/wt^;PDGFRα-CreER, RNF220-icHet*; *RNF220^flox/flox^;PDGFRα-CreER, RNF220-icKO*). The *RNF220-icKO* mice, as well as the two controls, were treated with tamoxifen to induce RNF220 deletion at different developmental stages. At first, the *RNF220-icKO* mice were treated with tamoxifen on P0 and P1 to induce RNF220 deletion from birth, and the brain samples were harvested on P3 after BrdU injection for 2 hours (fig. S5A). Double immunostaining confirmed RNF220 loss in Sox10^+^ cells in the corpus callosum (fig. S5A), and it was found that the number of proliferated Sox10^+^BrdU^+^ (*iWT*: 0.74 ± 0.34 × 10^2^/mm^2^; *icKO*: 0.36 ± 0.22 × 10^2^/mm^2^) or PDGFRα^+^Ki67^+^ (*iWT*: 3.08 ± 0.43 × 10^2^/mm^2^; *icKO*: 1.91 ± 0.38 × 10^2^/mm^2^) OPCs was severely decreased in *RNF220-icKO* mice (Fig. 2C), suggesting that RNF220 is involved in regulating OPC proliferation at the early developmental stage. Then tamoxifen was administrated to the *RNF220-icKO* mice from P8 to P10, and the brain samples were harvested, and OPC proliferation and OL differentiation were examined on P21 (Fig. 2D and fig. S5B). It was shown that there was no significant difference of the number of PDGFRα^+^Ki67^+^ OPCs between the *RNF220-iWT* and *RNF220-icKO* mice (fig. S5B), while the number of CC1^+^Sox10^+^ OLs was significantly less in the corpus callosum of *RNF220-icKO* brains (*iWT*: 11.33 ± 1.65 × 10^2^/mm^2^; *icKO*: 9.72 ± 1.60 × 10^2^/mm^2^; Fig. 2D), suggesting a direct differentiation defect during this later stage. Together, all these results indicate that RNF220 is involved in both two oligodendroglial developmental procedures of OPC proliferation and OL differentiation. Together, these results indicate that RNF220 is involved in regulating both OPC proliferation and differentiation to OL, and its deficiency impairs oligodendroglial development and leads to hypomyelination in mouse brains.

### OL lineage–specific RNF220 deficiency impairs learning and memory functions

**Fig. 3.**
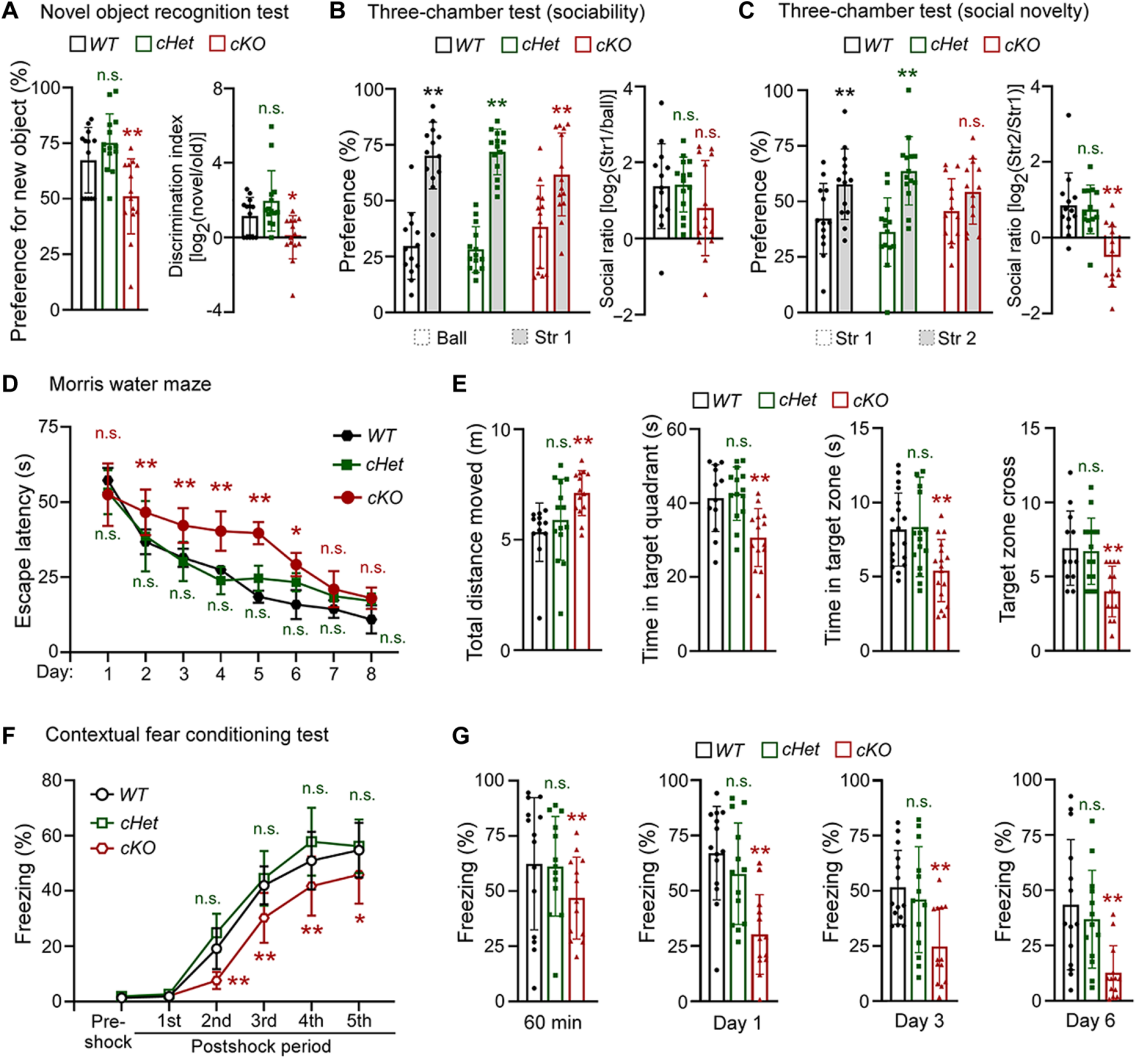
RNF220 deficiency in OL lineage cells causes impairment of learning and memory behaviors. Behavioral tests of novel object recognition (**A**), three-chamber sociability and social novelty (**B** and **C**), Morris water maze (**D** and **E**) and contextual fear conditioning (**F** and **G**) for P60 RNF220-WT (*n* = 15), RNF220-cHet (*n* = 15), and RNF220-cKO (*n* = 15) mice. (A) Bar graphs (mean ± SD) show the percentage of spending time close to new object and logarithm of discrimination index. (B) Bar graphs (mean ± SD) show the percentage of spending time close to inanimate ball and animated stranger mouse and logarithm of social ratio. (C) Bar graphs (mean ± SD) show the percentage of spending time close to new and familiar animated strangers and logarithm of social ratio. (D) and (E) Escape latencies (mean ± SD) to find the platform throughout the 8-day learning trials. (E) Spatial memory retrieval of these mice used in (D) was examined when the platform was removed, and bar graphs (mean ± SD) show total movement distances, time in target quadrant and zone, and cross for target zone. (F) and (G) Percentage of freezing behavior (mean ± SD) across fear conditioning sessions. (G) Fear memory of these mice used in (F) was tested by exposure to the environment only. Bar graphs (mean ± SD) show percentage of freezing behavior at 60 min, 1 day, 3 days, and 6 days after the contextual fear conditioning. Str, stranger. Statistical analyses are compared to respective control with Mann-Whitney *U* test with Bonferroni correction or unpaired Student’s *t* test. n.s. (not significant), *P* > 0.05; *P < 0.05; **P < 0.01.

It is known that myelin dysfunction has a profound effect on neurological functions including processing information during high cognition (*5*), and patients with RNF220 mutations have symptoms of intellectual disability (*16*, *17*). We next examined whether the RNF220 depletion–induced hypomyelination in *RNF220-cKO* mice would affect neural behaviors, particularly learning and memory. In the novel object recognition test, *RNF220-cKO* mice showed less preference to the new object as compared to *RNF220-WT* and *RNF220-cHet* littermates (preference for new object: *WT*: 67.36 ± 14.75%; *cHet*: 75.19 ± 12.98%; *cKO*: 51.09 ± 16.90%; discrimination index: *WT*: 1.15 ± 1.01; *cHet*: 1.97 ± 1.62; *cKO*: 0.02 ± 1.61; Fig. 3A), suggesting that nonspatial memory is impaired with RNF220 deficiency. During the three-chamber test that examines social interaction and memory, it was found that *RNF220-cKO* mice showed a significant preference for the animated stranger over the inanimate ball to the same extent as *RNF220-WT* and *RNF220-cHet* controls (preference: *WT*: 29.82 ± 14.93% for ball and 70.18 ± 14.93% for stranger 1; *cHet*: 28.17 ± 10.19% for ball and 71.83 ± 10.19% for stranger 1; *cKO*: 38.31 ± 18.48% for ball and 61.69 ± 18.48% for stranger 1; social ratio: *WT*: 1.38 ± 1.12; *cHet*: 1.42 ± 0.72; *cKO*, 0.80 ± 1.25; Fig. 3B). However, *RNF220-cKO* mice had a significant lower preference for the new stranger over the familiar stranger compared to the two control littermates (preference: *WT*: 42.31 ± 15.84% for stranger 1 and 57.69 ± 15.84% for stranger 2; *cHet*: 36.25 ± 15.35% for stranger 1 and 63.75 ± 15.35% for stranger 2; *cKO*: 45.61 ± 14.62% for stranger 1 and 54.39 ± 14.62% for stranger 2; social ratio: *WT*: 0.85 ± 0.86; *cHet*: 0.75 ± 0.64; *cKO*: −0.50 ± 0.80; Fig. 3C), suggesting an impairment of social memory.

The Morris water maze test was used to examine spatial learning and memory, and it was found that *RNF220-cKO* mice took more time to find the hidden platform on the second to sixth days during the 8-day training (escape latency: day 1: *WT*: 57.20 ± 4.12 s, *cHet*: 53.27 ± 7.34 s, *cKO*: 52.44 ± 10.42 s; day 2: *WT*: 36.80 ± 4.14 s, *cHet*: 38.66 ± 11.70 s, *cKO*: 46.58 ± 7.60 s; day 3: *WT*: 31.48 ± 2.99 s, *cHet*: 30.21 ± 6.49 s, *cKO*: 42.19 ± 5.76 s; day 4: *WT*: 27.45 ± 1.62 s, *cHet*: 23.93 ± 4.61 s, *cKO*: 40.37 ± 6.61 s; day 5: *WT*: 18.52 ± 2.04 s, *cHet*: 24.63 ± 4.20 s, *cKO*: 39.63 ± 3.68 s; day 6: *WT*: 15.91 ± 4.80 s, *cHet*: 23.33 ± 2.98 s, *cKO*: 29.21 ± 3.86 s; day 7: *WT*: 14.38 ± 2.96 s, *cHet*: 18.71 ± 1.77 s, *cKO*: 21.00 ± 6.03 s; day 8: *WT*: 10.94 ± 4.70 s, *cHet*: 17.11 ± 2.50 s, *cKO*: 18.06 ± 3.59 s; Fig. 3D), suggesting a higher escape latency and impairment of spatial learning. One day after the training trails, spatial memory was tested and it was shown that the total movement distance of *RNF220-cKO* mice was not shorter, even longer, than the *RNF220-WT* or *RNF220-cHet* control mice (*WT*: 534.20 ± 131.90 cm; *cHet*: 590.70 ± 184.00 cm; *cKO*: 711.30 ± 101.50 cm; Fig. 3E), suggesting that RNF220 knockout in OL lineage cells had no damaging effect on motor function. However, *RNF220-cKO* mice spent significantly less duration in both target quadrants (*WT*: 41.35 ± 9.05 s; *cHet*: 42.56 ± 7.22 s; *cKO*: 30.70 ± 7.82 s) and zones (*WT*: 8.91 ± 2.47 s; *cHet*: 7.71 ± 1.40 s; *cKO*: 4.69 ± 1.88 s) and had a severely decreased cross for target zone (*WT*: 6.92 ± 2.50; *cHet*: 6.71 ± 2.23; *cKO*: 4.00 ± 1.71) (Fig. 3E). Together, these results indicate that spatial learning and memory are impaired in the *RNF220-cKO* mice.

We next examined contextual fear memory using the fear conditioning test and found that *RNF220-cKO* mice showed reduced freezing performance in the postshock periods of the second to fifth trials during conditioning (freezing: preshock: *WT*: 1.38 ± 0.18%, *cHet*: 1.91 ± 0.68%, *cKO*: 1.83 ± 0.54%; first shock: *WT*: 1.93 ± 0.53%, *cHet*: 2.73 ± 0.22%, *cKO*: 2.11 ± 0.57%; second shock: *WT*: 19.19 ± 7.41%, *cHet*: 24.90 ± 6.89%, *cKO*: 7.65 ± 3.07%; third shock: *WT*: 42.01 ± 6.90%, *cHet*: 44.59 ± 9.92%, *cKO*: 30.31 ± 9.03%; fourth shock: *WT*: 50.94 ± 10.45%, *cHet*: 57.83 ± 12.25%, *cKO*: 41.74 ± 10.69%; fifth shock: *WT*: 54.78 ± 9.89%, *cHet*: 56.16 ± 9.64%, *cKO*, 45.91 ± 10.48%; Fig. 3F). Moreover, significant reduced freezing levels were observed in *RNF220-cKO* mice from short-term (1 hour) to long-term (6 days) after conditioning (60 min: *WT*: 62.38 ± 29.94%; *cHet*: 61.24 ± 22.54%; *cKO*: 46.86 ± 18.54%; 1 day: *WT*: 67.07 ± 21.17%; *cHet*: 57.70 ± 22.94%; *cKO*: 30.24 ± 17.97%; 3 days: *WT*: 51.66 ± 16.74%; *cHet*: 46.06 ± 24.04%; *cKO*: 24.67 ± 17.73%; and 6 days: *WT*: 43.47 ± 29.41%; *cHet*: 36.93 ± 22.11%; *cKO*: 12.72 ± 12.26%; Fig. 3G), suggesting that both short-term and remote memory are impaired. Collectively, these results indicate that RNF220 deficiency in OL lineage cells leads to leukodystrophy-like malfunction of learning and memory behaviors.

### RNF220 is essential for remyelination in adult brains

**Fig. 4.**
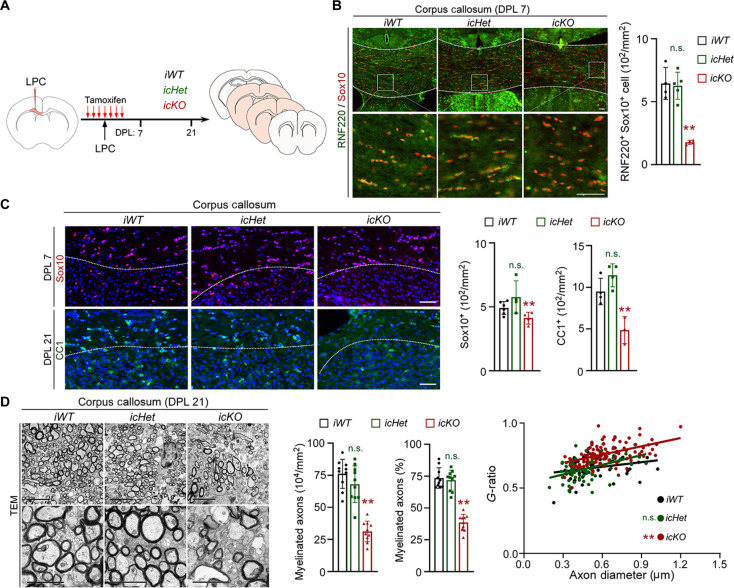
RNF220 knockout in OL lineage cells impairs remyelination in adult mouse brains. (**A**) Schematic diagram of l-α-lysophosphatidylcholine (LPC)–induced lesioning in the corpus callosum regions of *RNF220-iWT*, *RNF220-icHet*, and *RNF220-icKO* mice. (**B**) Immunofluorescence staining of RNF220 and Sox10 in the corpus callosum of *RNF220-iWT* (*n* = 3), *RNF220-icHet* (*n* = 3), and *RNF220-icKO* (*n* = 3) mice on DPL 7, and bar graphs (mean ± SD) show the quantification of RNF220^+^Sox10^+^ cells. Scale bars, 50 μm. (**C**) Immunofluorescence staining of Sox10 (on DPL 7) and CC1 (on DPL 21) in lesion areas of *RNF220-iWT* (*n* = 3), *RNF220-icHet* (*n* = 3), and *RNF220-icKO* (*n* = 3) mice brains, and bar graphs (mean ± SD) show the quantification of Sox10^+^ cells and CC1^+^ cells. Scale bars, 50 μm. (**D**) Electron microscopy images show the myelinated axons in lesion areas of *RNF220-iWT* (*n* = 4), *RNF220-icHet* (*n* = 4), and *RNF220-icKO* (*n* = 4) mice brains on DPL 21. Scale bars, 5 μm for upper panels and 2 μm for lower panels. Bar graphs (mean ± SD) show the quantification of number and percentage of myelinated axons, and scatterplots show *g*-ratio relative to axon diameters. Statistical analyses are compared to respective control with Mann-Whitney *U* test with Bonferroni correction. n.s. (not significant), *P* > 0.05; **P < 0.01.

Although the main signaling cascades and factors involved in the OL program of developmental myelination are shared by the remyelination program in adult (*22*), our recent work has identified an OPC-specific ubiquitin ligase that is only involved in remyelination (*23*). We next investigated whether RNF220 is required for OL regeneration and remyelination in adult mouse brains after demyelinating injury. To deplete RNF220 in adult OPCs, *RNF220-icKO* mice were administered with tamoxifen for 7 days to fully induce the expected recombination (Fig. 4A), which was reflected by a specific and marked decrease of RNF220 levels in the adult OPCs of *RNF220-icKO* mice (RNF220^+^Sox10^+^ cells: *iWT*: 6.45 ± 1.28 × 10^2^/mm^2^; *icHet*: 6.27 ± 1.08 × 10^2^/mm^2^; *icKO*: 1.78 ± 0.16 × 10^2^/mm^2^; Fig. 4B). On the fourth day, l-α-lysophosphatidylcholine (LPC) was injected into the corpus callosum for lesion induction, and the samples were harvested and examined on the day post lesion 7 (DPL 7) and DPL 21 (Fig. 4A). We first examined the population of OL lineage cells at lesion sites and found that the numbers of both Sox10^+^ OPCs on DPL 7 (*iWT*: 4.91 ± 5.06 × 10^2^/mm^2^; *icHet*: 5.77 ± 1.26 × 10^2^/mm^2^; *icKO*: 4.12 ± 0.46 × 10^2^/mm^2^; Fig. 4C) and CC1^+^ matured OLs on DPL 21 (*iWT*: 9.50 ± 1.58 × 10^2^/mm^2^; *icHet*: 11.44 ± 1.38 × 10^2^/mm^2^; *icKO*: 4.87 ± 1.62 × 10^2^/mm^2^; Fig. 4C) were decreased significantly in the brains of *RNF220-icKO* mice, compared to *RNF220-iWT* and *RNF220-icHet* littermates. Consistently, it was shown that on DPL 21 the number (*iWT*: 76.12 ± 11.30 × 10^4^/mm^2^; *icHet*: 68.05 ± 14.31 × 10^4^/mm^2^; *icKO*: 31.24 ± 8.05 × 10^4^/mm^2^; Fig. 4D) and percentage of remyelinated axons (*iWT*: 73.47 ± 8.05%; *icHet*: 71.85 ± 7.84%; *icKO*: 38.59 ± 6.44%; Fig. 4D) and the thickness of axonal myelin sheath (*g*-ratios: *iWT*: 0.65 ± 0.07; *icHet*: 0.65 ± 0.08; *icKO*: 0.76 ± 0.08; Fig. 4D) were severely reduced in the corpus callosum of *RNF220-icKO* mice relative to mice of the other two genotypes (Fig. 4D), suggesting an impairment of remyelination process. Together, these data indicate that RNF220 plays a crucial role for OL lineage cell regeneration in the context of white matter injury.

### RNF220 targets Olig1 and Olig2 for regulating their K63-linked ubiquitination

To identify the underlying mechanism by which RNF220 regulates OPC proliferation and OL differentiation, we decided to examine whether and how RNF220 could possibly modulate master TFs of transcriptional regulatory network during oligodendroglial development. To this end, coimmunoprecipitation (co-IP) assays in human embryonic kidney (HEK) 293 cells were used to test whether RNF220 directly interacted with key TFs, including Olig1, Olig2, Sox10, Nkx2-2, Hes5, Id2, Id4, Sox5, Sox6, and Ascl1 (*24*). Our results showed that among these TFs, only Ascl1, Olig1 and Olig2 were found to be interacted with RNF220 (fig. S6, A to J). Because neither the protein level nor the ubiquitination of Ascl1 was affected by RNF220 depletion in OPCs (fig. S6, K and L), we decided to exclude Ascl1 in subsequent investigations.

**Fig. 5.**
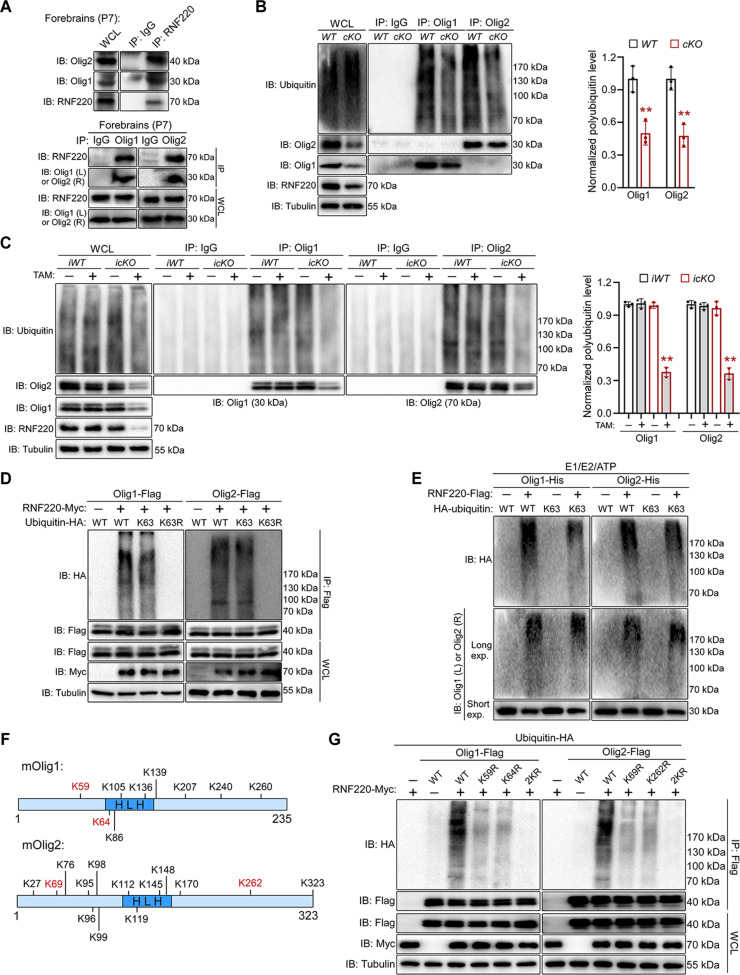
RNF220 regulates K63-linked polyubiquitination of Olig1 and Olig2. (**A**) Co-IP analyses of the interaction between endogenous RNF220 and Olig1 or Olig2 in forebrains with the indicated antibodies. IgG, immunoglobulin G. (**B**) Ubiquitination analyses of polyubiquitinated Olig1 and Olig2 in the forebrains of P7 *RNF220-WT* (*n* = 3) and *RNF220-cKO* (*n* = 3) mice, and bar graphs (mean ± SD) show normalized polyubiquitination levels against respective expression in the WT controls. (**C**) Analyses of ubiquitination levels of Olig1 and Olig2 in the forebrains of P60 *RNF220-iWT* (*n* = 3) and *RNF220-icKO* (*n* = 3) mice with tamoxifen treatment or not, and bar graphs (mean ± SD) show normalized polyubiquitination levels against respective expression in the WT controls without tamoxifen treatment. (**D**) Ubiquitination analyses of RNF220-mediated polyubiquitination of Olig1 and Olig2 in the presence of WT or indicated ubiquitin mutants in HEK293 cells. HA, hemagglutinin. (**E**) Ubiquitination analyses of RNF220-mediated polyubiquitination of Olig1 and Olig2 with purified proteins as indicated in vitro. (**F**) Schematic diagram showing all lysine sites in mouse Olig1 and Olig2 proteins and the lysine sites targeted by RNF220 are highlighted in red. (**G**) Ubiquitination analyses of RNF220-mediated polyubiquitination of WT or Olig1/2 mutants in HEK293 cells as indicated. IP, immunoprecipitation; WCL, whole-cell lysate; TAM, tamoxifen. Statistical analyses are compared to respective control with Mann-Whitney *U* test with Bonferroni correction in (D) and unpaired Student’s *t* test in (F). n.s. (not significant), *P* > 0.05; **P < 0.01.

Interaction between RNF220 and the Olig proteins was further confirmed by the reverse in vitro co-IP analyses (fig. S6M). In the forebrain lysates of P7 mice, it was found that RNF220 and Olig proteins were endogenously interacting (Fig. 5A). These results suggest that Olig1 and Olig2 are putative regulatory substrates of RNF220 during oligodendroglial development. As RNF220 is an E3 ubiquitin ligase involved in neural development (*25*), we then examined whether the ubiquitination of Olig proteins is regulated by RNF220. Through in vitro ubiquitination assays in HEK293 cells, it was found that the levels of polyubiquitinated Olig1 and Olig2 were both increased significantly by coexpression of RNF220 (fig. S7A), and this regulation depends on RNF220’s E3 ubiquitin ligase activity because either RNF220^ΔRING^ or RNF220^W539R^, which lacked the RING domain or had the essential site mutation respectively, failed to promote the polyubiquitination of Olig1 or Olig2 (fig. S7A). Moreover, when the endogenous Olig proteins were immunoprecipitated from P7 mice forebrains, their polyubiquitination levels in *RNF220-cKO* mice were markedly decreased compared to *RNF220-WT* and *RNF220-cHet* (Olig1: *WT*: 1.00 ± 0.12, *cKO*: 0.50 ± 0.11; Olig2: *WT*: 1.00 ± 0.10, *cKO*: 0.48 ± 0.10; Fig. 5B). In addition, the ubiquitinated Olig1 and Olig2 in adult forebrains were also significantly reduced when *RNF220-icKO* mice were treated with tamoxifen to deplete RNF220 [Olig1: *iWT* (TAM−): 1.00 ± 0.02, *iWT* (TAM+): 1.01 ± 0.04, *icKO* (TAM−): 0.99 ± 0.03, *icKO* (TAM+): 0.38 ± 0.04; Olig2: *iWT* (TAM−): 1.00 ± 0.03, *iWT* (TAM+): 0.98 ± 0.03, *icKO* (TAM−): 0.96 ± 0.06, *icKO* (TAM+): 0.36 ± 0.05; Fig. 5C]. Together, these results suggest that Olig1 and Olig2 are direct ubiquitination substrates for the E3 ubiquitin ligase RNF220 in OL lineage cells.

Various types of ubiquitination linkages have distinct regulatory effects on protein stability and activity (*26*). To determine the type of ubiquitin chain added by RNF220 on Olig proteins, different and specific ubiquitin mutants were used in the ubiquitination assays in HEK293 cells. It was shown that both Olig1 and Olig2 were only ubiquitinated by RNF220 when K63-type ubiquitin was present (fig. S7B). Consistent with this, when the K63R mutated ubiquitin was coexpressed, the RNF220 induced polyubiquitination levels of Olig proteins were fully diminished (Fig. 5D). When an in vitro ubiquitination assay was carried out using purified proteins, it was found that RNF220 promoted polyubiquitination of the Olig proteins efficiently in the presence of wild-type (WT) or K63 ubiquitin (Fig. 5E). Lysine residues in substrate act as the stereotypical ubiquitination site(s), and there are 8 and 14 lysine residues in mouse Olig1 and Olig2 proteins, respectively (Fig. 5F). To determine the responsible ubiquitination site(s), we individually mutated all these lysines into arginines and then examined RNF220-mediated ubiquitination of each mutant. It was found that K59/K64 and K69/K262 were required for the polyubiquitination of Olig1 and Olig2, respectively (fig. S7, C and D). Furthermore, when these lysine residues were simultaneously mutated into arginines, RNF220 failed to enhance the polyubiquitination levels of the resulted Olig1^2KR^ and Olig2^2KR^ mutants (Fig. 5G), suggesting that these lysine residues are direct ubiquitination sites. Together, these results indicate that RNF220 regulates Olig1 and Olig2 ubiquitination by adding K63-linked polyubiquitin chains at the lysine sites of K59/K64 and K69/K262, respectively.

### RNF220-mediated ubiquitination of Olig1 and Olig2 maintains the protein stability

**Fig. 6.**
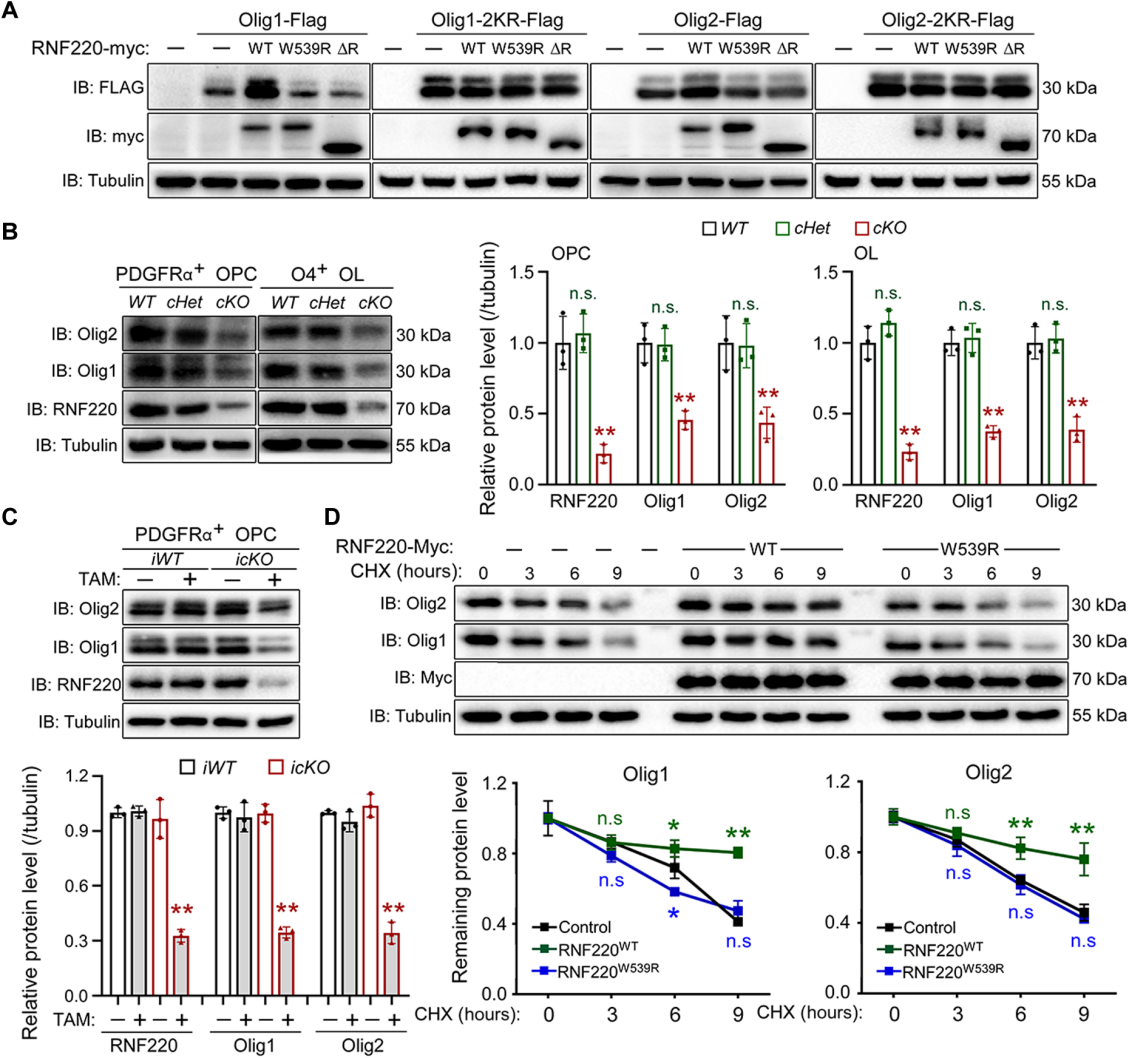
RNF220 maintains Olig1 and Olig2 proteins stabilization through regulating their ubiquitination. (**A**) WB analyses of protein expression of WT or Olig1/2 mutants when coexpressed with WT RNF220 or mutants lacking ubiquitin E3 ligase activity in HEK293 cells. (**B**) WB analyses of endogenous protein expression of RNF220, Olig1, and Olig2 in OPC and OL cells, isolated as in fig. S1A, from the brains of P7 *RNF220-WT* (*n* = 3), *RNF220-cHet* (*n* = 3), and *RNF220-cKO* (*n* = 3) mice, and bar graphs (mean ± SD) show normalized levels against respective protein expression in the WT controls. (**C**) WB analyses of endogenous protein expression of RNF220, Olig1, and Olig2 in isolated OPC from the brains of P60 *RNF220-iWT* (*n* = 3) and *RNF220-icKO* (*n* = 3) mice with tamoxifen treatment or not, and bar graphs (mean ± SD) show normalized levels against respective protein expression from the WT control without tamoxifen treatment. (**D**) Cycloheximide chase analyses of protein half-lives of endogenous Olig1 and Olig2 in MOPC cells overexpressing WT RNF220 or RNF220^W539R^ mutant lacking E3 ligase activity, and broken line graphs (mean ± SD) show normalized levels against respective protein expression from the control without cycloheximide treatment. ΔR, ΔRING; CHX, cycloheximide. Statistical analyses are compared to respective control with Mann-Whitney *U* test with Bonferroni correction. n.s. (not significant), *P* > 0.05; *P < 0.05; **P < 0.01.

After determining that RNF220 targets Olig proteins for ubiquitination during oligodendroglial development, we next examined whether RNF220 is involved in controlling their protein quality. Unexpectedly, it was found that the protein levels of both Olig1 and Olig2 were increased markedly by coexpression of RNF220 in HEK293 cells (Fig. 6A and fig. S8A). Moreover, this regulation was diminished when either RNF220 mutants lacking the E3 ligase activity, or Olig1 and Olig2 mutants lacking the essential ubiquitination residues, were used for the analyses (Fig. 6A and fig. S8A). Similarly, RNF220 knockdown significantly down-regulated Olig1 and Olig2 protein levels in MOPC cells, while their mRNA expression was not changed by RNF220 siRNAs (fig. S8, B and C). Consistent with these results, the protein levels of both Olig1 and Olig2 were significantly reduced in the respective isolated OPCs or OLs when RNF220 was depleted in *RNF220-cKO* (OPC: RNF220: *WT*: 1.00 ± 0.18, *cHet*: 1.07 ± 0.14, *cKO*: 0.22 ± 0.07; Olig1: *WT*: 1.00 ± 0.14, *cHet*: 0.99 ± 0.11, *cKO*: 0.46 ± 0.07; Olig2: *WT*: 1.00 ± 0.19, *cHet*: 0.98 ± 0.16, *cKO*, 0.44 ± 0.11; OL: RNF220: *WT*: 1.00 ± 0.12, *cHet*: 1.14 ± 0.09, *cKO*: 0.23 ± 0.06; Olig1: *WT*: 1.00 ± 0.09, *cHet*: 1.03 ± 0.10, *cKO*: 0.38 ± 0.04; Olig2: *WT*: 1.00 ± 0.11, *cHet*: 1.03 ± 0.10, *cKO*: 0.39 ± 0.09; Fig. 6B) or *RNF220-icKO* mice [RNF220: *iWT* (TAM−): 1.00 ± 0.03, *iWT* (TAM+): 1.01 ± 0.03, *icKO* (TAM−): 0.97 ± 0.11, *icKO* (TAM+): 0.33 ± 0.03; Olig1: *iWT* (TAM−): 1.00 ± 0.03, *iWT* (TAM+): 0.97 ± 0.08, *icKO* (TAM−): 1.00 ± 0.05, *icKO* (TAM+): 0.35 ± 0.03; Olig2: *iWT* (TAM−): 1.00 ± 0.01, *iWT* (TAM+): 0.95 ± 0.05, *icKO* (TAM−): 1.04 ± 0.06, *icKO* (TAM+): 0.34 ± 0.06; Fig. 6C]. All these results suggest that this RNF220-mediated K63-linked polyubiquitination of Olig1 and Olig2 is involved in promoting their protein stability. To further confirm this conclusion, the protein degradation kinetics of endogenous Olig1 and Olig2 were examined in MOPC cells through cycloheximide chase analysis. It was shown that overexpressing RNF220 increased the stabilities of Olig proteins and this modulation was depended on its E3 ubiquitin ligase activity, as the ligase-dead mutant lost the regulatory capability (Olig1: Control: 0 hours: 1.00 ± 0.10, 3 hours: 0.87 ± 0.04, 6 hours: 0.72 ± 0.06, 9 hours: 0.41 ± 0.01; RNF220^WT^: 0 hours: 1.00 ± 0.02, 3 hours: 0.86 ± 0.04, 6 hours: 0.83 ± 0.05, 9 hours, 0.80 ± 0.03; RNF220^W539R^: 0 hours: 1.00 ± 0.03, 3 hours: 0.79 ± 0.04, 6 hours: 0.58 ± 0.01, 9 hours: 0.47 ± 0.06; Olig2: Control: 0 hours: 1.00 ± 0.03, 3 hours: 0.87 ± 0.04, 6 hours: 0.64 ± 0.02, 9 hours: 0.46 ± 0.05; RNF220^WT^: 0 hours: 1.00 ± 0.04, 3 hours: 0.91 ± 0.03, 6 hours: 0.82 ± 0.06, 9 hours: 0.76 ± 0.09; RNF220^W539R^: 0 hours: 1.00 ± 0.03, 3 hours: 0.84 ± 0.06, 6 hours: 0.62 ± 0.05, 9 hours: 0.42 ± 0.01; Fig. 6D). Conversely, RNF220 knockdown decreased the stabilities of both Olig1 and Olig2 proteins (fig. S8D). Together, these results indicate that RNF220 maintains the stability of Olig proteins by regulating their K63-linked polyubiquitination in OL lineage cells. To further examine the RNF220 regulation of Olig1 and Olig2 during oligodendroglial development, we coexpressed Olig1 or Olig2 with RNF220 siRNA in MOPC cells and found that Olig2 or Olig1 overexpression rescued the expression levels of OPC markers (fig. S9A) or OL markers after T3-induced differentiation respectively (fig. S9B), while it had no effect on RNF220 expression, suggesting that Oligo proteins could reverse RNF220 loss-caused impairment of the proliferation and differentiation of oligodendroglial cells. This indicates that they are the main downstream effectors of RNF220 during oligodendroglial development.

### Leukodystrophy-related mutations impair RNF220 regulation on Olig proteins stabilization, oligodendroglial development, and myelination

**Fig. 7.**
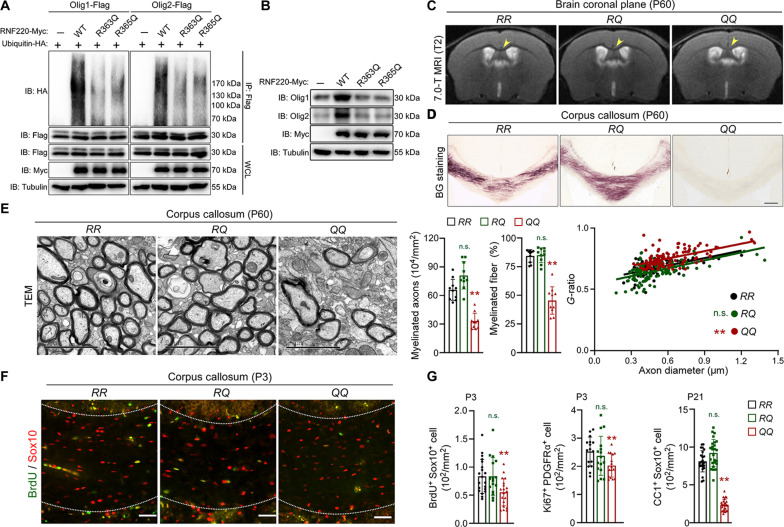
Leukodystrophy-related mutations impair RNF220 regulation on Olig protein stabilization, oligodendroglial differentiation, and myelination. (**A**) Ubiquitination analyses of polyubiquitinated Olig1 and Olig2 when coexpressed with WT RNF220 or leukodystrophy-related missense mutants, RNF220^R363Q^ and RNF220^R365Q^, in HEK293 cells. (**B**) WB analyses of endogenous Olig1 and Olig2 expression in MOPC cells overexpressing WT RNF220 or leukodystrophy-related missense mutants, RNF220^R363Q^ and RNF220^R365Q^. (**C**) Representative T2-weighted mouse brain MRI scanning of P60 *RNF220-RR* (*n* = 3), *RNF220-RQ* (*n* = 3), and *RNF220-QQ* (*n* = 3) mice. The arrows indicate the corpus callosum. (**D**) Representative images showing myelin staining (BG) in corpus callosum regions of P60 *RNF220-RR* (*n* = 3), *RNF220-RQ* (*n* = 3), and *RNF220-QQ* (*n* = 3) mice. (**E**) Electron microscopy images of the corpus callosum transverse sections from P60 *RNF220-RR* (*n* = 4), *RNF220-RQ* (*n* = 4), and *RNF220-QQ* (*n* = 4) mice. Scale bars, 2 μm. Bar graphs (mean ± SD) show quantification of the number and percentage of myelinated axons, and scatterplots show *g*-ratio relative to axon diameter. (**F**) Immunofluorescence staining assays of BrdU and Sox10 in the corpus callosum regions of P3 *RNF220-RR* (*n* = 3), *RNF220-RQ* (*n* = 3), and *RNF220-QQ* (*n* = 3) mice. Scale bars, 50 μm. (**G**) Bar graphs (mean ± SD) show the quantification of BrdU^+^Sox10^+^ cells, Ki67^+^PDGFRα^+^ cells on P3, and CC1^+^Sox10^+^ cells on P21. Statistical analyses are compared to respective control with Mann-Whitney *U* test with Bonferroni correction or unpaired Student’s *t* test. n.s. (not significant), *P* > 0.05; **P < 0.01.

As the phenotypes observed in *RNF220-cKO* mice, including corpus callosum agenesis and impairment of learning and memory, are closely related to clinical symptoms of patients harboring RNF220 missense mutations (*16*, *17*), we wondered whether our findings may demonstrate an underlying pathological mechanism of leukodystrophy. Toward this goal, we first tried to investigate the effect of leukodystrophy-related mutations, RNF220^R363Q^ and RNF220^R365Q^, on Olig protein regulation. Co-IP analyses results showed that the two RNF220 mutations impaired its binding affinity with Olig1 and Olig2 (fig. S10A). Moreover, the capability to promote the polyubiquitination of Olig proteins was markedly reduced by either of these two missense variants (Fig. 7A and fig. S10B). Furthermore, both the two mutants failed to increase protein levels of Olig1 and Olig2 when coexpressed in HEK293 cells (fig. S10C). In addition, when overexpressed in MOPC cells, both RNF220^R363Q^ and RNF220^R365Q^ lost the ability to increase the protein levels of endogenous Olig1 and Olig2 (Fig. 7B and fig. S10D). Together, these results indicate that leukodystrophy-related RNF220 mutations impair its regulation of ubiquitination of Olig1 and Olig2 and their protein stability.

To study the RNF220 neuropathological mutations on oligodendroglial differentiation and myelination, we generated a RNF220^R365Q^ knock-in mouse model through CRISPR-Cas9 technology (fig. S11A). The cross resulted in WT, heterozygous, and homozygous mice, which we will hereafter refer to as *RNF220-RR*, *RNF220-RQ*, and *RNF220-QQ*, respectively. It was found that both the mRNA and protein levels of RNF220 in forebrain OPC and OL cells were comparable among the three mouse lines (fig. S11, B and C). There was no difference in fertility and survival rate among these three genotypes. Moreover, they showed no significant difference in appearance, body weight, brain weight, and structures at adult age (fig. S11, D to F).

T2-weighted MRI imaging analysis was first used to study the myelin deposition in adult brains of the three mice, and it was shown that the signal intensity of the corpus callosum in *RNF220-QQ* mice, but not in *RNF220-RQ*, was higher than that in the *RNF220-RR* controls (Fig. 7C), suggesting that there is corpus callosum agenesis in this disease mouse model. Consistent with this finding, BG staining and TEM analyses showed that the axonal fibers of corpus callosum in *RNF220-QQ* mice were severely hypomyelinated (the number of myelinated axons: *RR*: 65.92 ± 11.07 × 10^4^/mm^2^; *RQ*: 81.05 ± 14.27 × 10^4^/mm^2^; *QQ*: 33.01 ± 8.44 × 10^4^/mm^2^; the percentage of myelinated axons: *RR*: 84.39 ± 5.52%; *RQ*: 85.01 ± 6.39%; *QQ*: 45.44 ± 12.01%; *g*-ratios: *RR*: 0.67 ± 0.06; *RQ*: 0.65 ± 0.08; *QQ*: 0.76 ± 0.07; Fig. 7, D and E, and fig. S12, A and B). Furthermore, the mRNA and protein levels of myelin-related molecules, including MBP, PLP, MAG, and MOG, were significantly down-regulated in the forebrains of *RNF220-QQ* mice (fig. S12, C to E). When proliferation of OPC was examined in the corpus callosum of P3 mice, it was found that the number of either BrdU^+^Sox10^+^ (*RR*: 0.82 ± 0.25 × 10^2^/mm^2^; *RQ*: 0.84 ± 0.33 × 10^2^/mm^2^; *QQ*: 0.54 ± 0.21 × 10^2^/mm^2^; Fig. 7G) or Ki67^+^PDGFRα^+^ (*RR*: 2.52 ± 0.51 × 10^2^/mm^2^; *RQ*: 2.37 ± 0.69 × 10^2^/mm^2^; *QQ*: 2.03 ± 0.41 × 10^2^/mm^2^; Fig. 7G) cells was decreased in the homozygous knock-in mice, compared to heterozygous and WT mice (Fig. 7, F and G, and fig. S12F). Moreover, in P21 mouse corpus callosum, the number of CC1^+^Sox10^+^ mature OLs (*RR*: 8.13 ± 1.41 × 10^2^/mm^2^; *RQ*: 9.21 ± 1.80 × 10^2^/mm^2^; *QQ*: 2.36 ± 0.75 × 10^2^/mm^2^; Fig. 7G) was also reduced in the *RNF220-QQ* mice (Fig. 7G and fig. S12F), implying an OL differentiation defect. Together, these results indicate that the oligodendroglial development and myelination are impaired by the leukodystrophy-related RNF220 missense mutation.

**Fig. 8.**
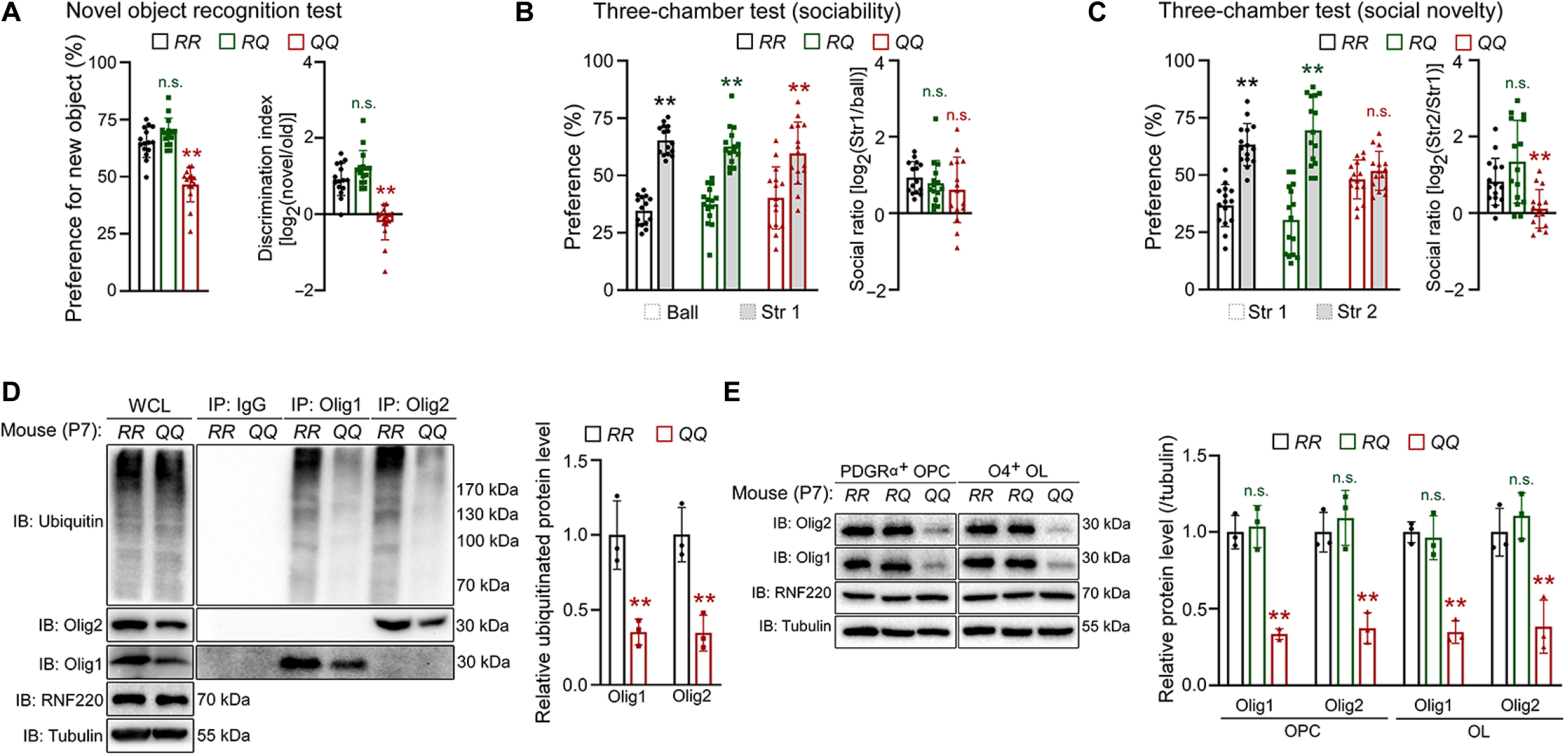
Leukodystrophy-related RNF220 mutation knock-in mice show impaired regulation of learning and memory behaviors and of Olig proteins. (**A** to **C**) Behavioral tests of novel object recognition (A), three-chamber sociability, and social novelty [(B) and (C)], for P60 *RNF220-RR* (*n* = 15), *RNF220-RQ* (*n* = 15), and *RNF220QQ* (*n* = 15) mice. (A) Bar graphs (mean ± SD) show the percentage of spending time close to new object and logarithm of discrimination index. (B) Bar graphs (mean ± SD) show the percentage of spending time close to inanimate ball and animated stranger mouse and logarithm of social ratio. (C) Bar graphs (mean ± SD) show the percentage of spending time close to new and familiar animated strangers and logarithm of social ratio. (**D**) Ubiquitination analyses of polyubiquitinated Olig1 and Olig2 in the forebrains of P7 *RNF220-RR* (*n* = 3) and *RNF220-QQ* (*n* = 3) mice, and bar graphs (mean ± SD) show normalized polyubiquitination levels against respective expression in the WT controls. (**E**) WB analyses of endogenous protein expression of Olig1 and Olig2 in OPC and OL cells, isolated as in fig. S1A, from the brains of P7 *RNF220-RR* (*n* = 3), *RNF220-RQ* (*n* = 3), and *RNF220QQ* (*n* = 3) mice, and bar graphs (mean ± SD) show normalized levels against respective protein expression in the WT controls. Statistical analyses are compared to respective control with Mann-Whitney *U* test with Bonferroni correction or unpaired Student’s *t* test. n.s. (not significant), *P* > 0.05; **P < 0.01.

We then used the above neural behavioral tests to examine the learning and memory abilities of these three mouse lines. Similar to *RNF220-cKO* mice, *RNF220-QQ* mice showed abnormal performances in tests of new object recognition (preference for new object: *RR*: 65.10 ± 6.65%; *RQ*: 69.35 ± 6.24%; *QQ*: 46.60 ± 7.60%; discrimination index: *RR*: 0.92 ± 0.42; *RQ*: 1.21 ± 0.47; *QQ*: −0.21 ± 0.47; Fig. 8A), three-chamber social interaction (preference: *RR*: 34.59 ± 6.38% for ball and 65.41 ± 6.38% for stranger 1; *RQ*: 37.36 ± 8.61% for ball and 62.64 ± 8.61% for stranger 1; *QQ*: 40.26 ± 13.51% for ball and 59.74 ± 13.51% for stranger 1; social ratio: *RR*: 0.94 ± 0.41; *RQ*: 0.78 ± 0.60; *QQ*: 0.62 ± 0.86; Fig. 8B and preference: *RR*: 36.69 ± 9.19% for stranger 1 and 63.31 ± 9.19% for stranger 2; *RQ*: 30.44 ± 14.71% for stranger 1 and 69.56 ± 14.71% for stranger 2; *QQ*: 48.09 ± 8.51% for stranger 1 and 51.91 ± 8.51% for stranger 2; social ratio: *RR*: 0.82 ± 0.61; *RQ*: 1.34 ± 1.01; *QQ*: 0.12 ± 0.50; Fig. 8C), Morris water maze (fig. S12, G and H) and fear conditioning (fig. S12, I and J), suggesting that the homozygous RNF220^R365Q^ mouse model has neuropathological cognition deficits. Last, when the levels of Olig1 and Olig2 were examined in *RNF220-QQ* mouse brains, we found that the polyubiquitination levels of Olig proteins were remarkably decreased in their forebrains compared with the WT littermates (Olig1: *RR*: 1.00 ± 0.23, *QQ*: 0.35 ± 0.09; Olig2: *RR*: 1.00 ± 0.18, *QQ*: 0.35 ± 0.12; Fig. 8D). Consistently, the proteins levels of both Olig1 and Olig2 in the isolated OPC and OL cells from P7 *RNF220-QQ* mouse forebrains were much lower than that of the controls (OPC: Olig1: *RR*: 1.00 ± 0.11, *RQ*: 1.04 ± 0.14, *QQ*: 0.33 ± 0.03; Olig2: *RR*: 1.00 ± 0.13, *RQ*: 1.09 ± 0.18, *QQ*: 0.37 ± 0.10; OL: Olig1: *RR*: 1.00 ± 0.07, *RQ*, 0.96 ± 0.14, *QQ*, 0.35 ± 0.07; Olig2: *RR*: 1.00 ± 0.16, *RQ*: 1.11 ± 0.15, *QQ*: 0.38 ± 0.17; Fig. 8E), suggesting dysfunction of the maintenance of Olig proteins stability. Together, all these results suggest that RNF220 mutations impair the regulation of Olig proteins stability and oligodendroglial development, which can serve as a potential etiological mechanism for leukodystrophy pathogenesis.

## DISCUSSION

In this study, we found that RNF220 promotes K63-linked polyubiquitination of Olig1 and Olig2, two master regulatory TFs involved in oligodendroglial development. RNF220 regulated K63-linked polyubiquitination to maintain Olig1 and Olig2 protein stability, and this modulation is required for oligodendroglial development and myelination, as well as remyelination. Moreover, in the pathomimetic RNF220^R365Q^ knock-in mouse model, deregulation of ubiquitination and stabilization of the Olig proteins leads to maldevelopment of oligodendroglia, corpus callosum agenesis, and leukodystrophy-like symptoms.

Olig1 and Olig2 are key TFs of the gene regulatory network that control oligodendroglial development and myelination in the brain (*24*), and their stage-specific functions heavily depends on posttranslational modifications, such as phosphorylation and acetylation. During neural development, Olig2 phosphorylation is dynamically regulated, and phosphorylation of the triple serine residues at the N terminus is required for neural progenitor proliferation (*27*) while its phosphorylation at Ser^147^ in the basic helix-loop-helix (bHLH) domain is crucial for motor neuron differentiation in spinal cord (*10*). Moreover, Ser^147^ dephosphorylation of Olig2 is required for later OPC fate determination (*10*). During OL maturation, Olig1 protein is regulated by acetylation and phosphorylation, which are essential for its translocation from the nuclear to the cytoplasm and subsequent developmental processes (*8*, *9*). Here, we find that in OL lineage cells, Olig proteins have undergone K63-linked polyubiquitination for stabilization, which is involved in regulating both OPC proliferation and OL differentiation. Moreover, we found that this ubiquitination has no effect on Olig1 subcellular localization as we found that the percentage of HEK293 cells with Olig1 cytoplasm distribution was not changed by overexpressing WT RNF220 or its E3 ubiquitin ligase–dead mutants (fig. S13). Therefore, the RNF220-mediated ubiquitination of Olig1 is an independent regulatory process from those modifications of acetylation or phosphorylation. Although we provide evidence that the protein levels of Olig1 and Olig2 are enhanced by this K63-linked ubiquitination, we cannot be certain whether it is also involved in regulating the interactome and transcriptional activities of Olig proteins, which the phosphorylation and SUMOylation for Olig2 is involved in (*10*, *27*, *28*). It would be interesting to further study whether there is any cross-talk among these PTMs to regulate Olig proteins activities during oligodendroglial development and (re)myelination.

There are seven internal lysine residues in the ubiquitin protein, and accordingly, seven different kinds of polyubiquitin chains can form, thereby conferring differential fates to a single substrate (*29*, *30*). Both K48 ubiquitination–induced degradation and nonproteolytic K63 ubiquitination play crucial roles during neural development and for modulating brain functions, as well as in the pathology neurological diseases (*31*, *32*). As an E3 ubiquitin ligase, RNF220 is capable of adding such distinct polyubiquitin chains to exert its biological functions. Our previous studies have found that it modulates K63-linked ubiquitination of Gli protein to fine-tune Shh signaling activity during spinal cord patterning (*18*) as well as K48-linked ubiquitination of α-amino-3-hydroxy-5-methyl-4-isoxazole propionic acid type glutamate receptors (AMPARs) to regulate synaptic transmission and plasticity in forebrain excitatory neurons (*33*). Here, we further uncover a novel role in OL lineage cells where RNF220 regulates K63-linked ubiquitination of Olig proteins to promote their stabilization for (re)myelination. However, it is unknown how RNF220 achieves such specificity in different neural cells. As the Olig protein levels are decreased markedly when RNF220 is lacking, further efforts are also needed to find out the underlying E3 ubiquitin ligase(s) regulating their K48-linked ubiquitination and protein degradation. Moreover, it would be interesting to determine how the different activities of these E3 ubiquitin ligases are balanced to bidirectionally modulate Olig1 and Olig2 expression levels for ensuring normal oligodendroglial development and (re)myelination.

The E3 ubiquitin ligase–mediated ubiquitination is a fundamental PTM and participates in various processes of neural development, from neural progenitor proliferation and differentiation to synapse formation and circuit connection (*32*, *34*). Most of our knowledge about the pathological roles of ubiquitination-controlled proteins homeostasis is based on the identified E3 ubiquitin ligase mutations in neurological disorders. Of the known and putative E3 ubiquitin ligase genes in human genome, around 13% have been found to be mutated in common and rare neurological disorders, several of which are attributed to deregulation of neural development, including autism spectrum disorder, Angelman syndrome, and Gordon Holmes syndrome (*14*). However, among these mutated E3 ubiquitin ligases, only a few have been extensively studied to understand their physiological roles and pathological mechanisms, such as in UBE3A, PARKIN, etc. (*14*). HLD is a developmental disease caused by deregulation of oligodendroglial development and myelination. Among the identified HLD-related genes in the OMIM database, only *RNF220* and *VPS11* may encode putative E3 ubiquitin ligases (*15*). VPS11 is a subunit of the HOPS/CORVET complex for endosomal fusion, and recently, it has been reported to function as an E3 ubiquitin ligase involved in fine-tuning various signaling pathways, including Wnt, estrogen receptor α, and nuclear factor κB (*35*). Although a recent study has found that VPS11 is expressed in mature OLs and may be involved in myelination (*36*), more efforts are needed to investigate whether the VPS11-related HLD depends on its ubiquitin ligase activity. In this study, we present ample evidence that dysregulation of RNF220-regulated ubiquitination and stabilization of Olig proteins are implicated in the pathology of leukodystrophy. Furthermore, we and other groups have recently found several E3 ubiquitin ligases, including Cbl, Nedd4, and RNF43, that are involved in oligodendroglial differentiation and (re)myelination (*23*, *37*, *38*), though genetic epidemiology studies have so far not found any pathological mutation in these genes in patients with HLD. The prevalence of E3 ubiquitin ligase–mediated proteostasis in OPC and OL cells suggests that uncovering the underlying regulatory network of ubiquitination will be helpful in furthering our understanding of the etiology of leukodystrophy.

Although the *RNF220-QQ* mouse is a global knock-in model that faithfully mimics the scenario in patients, *RNF220-QQ* animals do not recapitulate all the features in patient homozygous for RNF220 mutations. For example, *RNF220-QQ* mice showed no significant difference in survival and walking performance during our 12-month observation, while most affected patients suffered from severe ataxia during their early teenage years and died at their second teenage years (*16*, *17*). As it was shown that the total swimming distance in *RNF220-cKO* (Fig. 3E) or *RNF220-QQ* mice (fig. S12H) was not shorter than their control mice during the Morris water maze tests, we think that RNF220 might not be required for movement regulation. However, in our following rotarod test, unexpectedly, it was found that the *RNF220-cKO* and *RNF220-QQ* mice exhibited shorter latency to fall from a uniformly accelerating apparatus compared with the respective control littermates (fig. S14), suggesting that RNF220 depletion or mutation in oligodendroglial cells indeed impairs mice motor ability. Therefore, RNF220-mediated myelination is involved in regulating fine-tune motor but not general movement. The differences in neuropathology between rodent and human, as well as the complicated motor behaviors we have found, indicate that more accurate disease mice models are required to dissect the distinct pathogenic reasons for the complex neuropsychiatry and neurodevelopmental disorders. Moreover, it would be helpful by constructing and investigating larger animal model, such as no-human primate, of which brain is more to our human being.

## MATERIALS AND METHODS

### Cell culture and transfection

Human HEK293 cells were grown in Dulbecco’s modified Eagle’s medium (DMEM) (Gibco, 11965092) supplemented with 10% fetal bovine serum (Gibico, A5669701), and penicillin-streptomycin (100 mg/ml) (Biological Industries, 03-031-1B). The Mouse OL precursor cell (MOPC) line was generated by immortalization of the primary mouse OPC cells isolated from mouse forebrains and we obtained it from the Conservation Genetics of CAS Kunming Cell Bank. MOPCs were cultured as previously described with minor modifications (*39*). MOPCs were grown in the basic growth medium [DMEM plus 0.1% BSA (Solarbio, A8020), apo-transferrin (50 μg/ml; Sigma-Aldrich, T1147), 1 mM sodium pyruvate (Gibico, 11360070), 30 nM sodium selenite (Sigma-Aldrich, S5261), 10 nM D-biotin (Sigma-Aldrich, 2031), 10 nM hydrocortisone (Sigma-Aldrich, 3867), PDGF-AA (10 ng/ml; Peprotech, 100-13A), basic fibroblast growth factor (10 ng/ml; Proteintech, 100-18B-100), and 2% N2 (Life Technologies, 17502-048)]. For differentiation, T3 (10 ng/ml; MedChemExpress, HY-A0070) was included in the growth medium. Both HEK293 and MOPC cells were transfected using Lipofectamine 2000 (Invitrogen, 11668019) according to the manufacturer’s instructions.

### Animal procedures

All mice with C57BL/6 background were maintained and handled according to guidelines approved by the Animal Care and Use Committee of the Kunming Institute of Zoology, Chinese Academy of Sciences (IACUC-PA-2022-02-022). The conditional RNF220 knockout mouse model *RNF220^flox/flox^* was used as previously described (*18*). *Olig1-Cre* (*19*) and *PDGFR*α*-CreER* (*21*) mice were used for the generation of OL lineage conditional and inducible knockout mouse model, respectively. *RNF220^R365Q^* knock-in founder mouse lines (strain no. T054604) were generated by NRCMM (National Resource Center for Mutant Mice, Nanjing University) using CRISPR-Cas9 technology. In brief, the sequences 5′-GATACGGGCCACCACACTCC-3′ and 5′-GAAGCCACCTTCCAGGAGTG-3′ were chosen as the single guide RNA targeting genomic DNA sequences. A donor vector containing the mutation of RNF220^R365Q^ flanked by sequences upstream and downstream of the genomic region was used to insert the specific mutation by homologous recombination.

All genotypes described were confirmed by PCR using genomic DNA prepared from tail tips. *RNF220* floxed allele was genotyped as previously described (*18*). The *Cre* allele was identified by the following primers: forward, 5′-GCCTGCATTACCGGTCGATGC-3′ and reverse, 5’-CAGGGTGTTATAAGCAATCCC-3′. *RNF220* KI allele was genotyped by real-time PCR using the following primers: forward, 5′-ATTGAGTGTATTTCACGGGAGCC-3′ and reverse, 5′-CAGCCTCACAATTCCATTTCCC-3′, supplemented with taqman probes: WT: 5′-(VIC)-CGGATACGGGCCACCACACTCCTG-(BHQ1)-3′, and KI: 5′-(FAM)-CGGATACAGGCCACCACATTGCTG-(BHQ1)-3′.

To induce *RNF220* knockout in adult *RNF220^flox/flox^;PDGFR*α*-CreER* mice, intragastrical administration of tamoxifen (Sigma-Aldrich, T5648) at a dose of 200 mg/kg body weight was given daily for 7 days continuously. The model of adult injury and remyelination was performed as described previously (*40*). LPC (1 μl; Sigma-Aldrich, L4219) of 1% solution was injected into the corpus callosum, at the coordinate: 0.8 mm lateral, 0.8 mm rostral to bregma, 1.2 mm deep to brain surface, to induce lesion on the fourth day of tamoxifen-administration. For BrdU pulse labeling, animals were injected intraperitoneally with BrdU (500 mg/kg; Sigma-Aldrich, B5002) per body weight at 2 hours before sacrifice.

### Plasmids and reagents

Ubiquitin constructs were gifts from Ceshi Chen’s lab. Olig2 expression plasmid was a gift from Y. Li’s laboratory (Shanghai Jiao Tong University School of Medicine). The construct containing mouse Olig1 cDNA was purchased from OriGene (MR203390). RNF220 constructs were used as previously described (*18*).

To prepare the Olig1 and Olig2 KR mutation constructs, site-directed mutagenesis was carried out by PCR-driven overlap extension using *Pfu* DNA polymerase (TianGen, EP101-02) as previously described (*41*). The following siRNAs were used to knock down RNF220 in MOPC cells: control siRNA: *siControl*, 5’-TTCTCCGAACGTGTCACGT-3′; siRNAs for RNF220: *siRNA-1#*: 5’-CCTGCAAGAACAGCGACATTG-3′, *siRNA-2#*: 5’-AGACTGAAGCACATGTAATAT-3′, and *siRNA-3#*: 5’-GGAGTATGGGAAACCACAATA-3′.

### RNA isolation, cDNA synthesis, and quantitive real-time PCR

Total RNA was isolated using TRIzol reagent (TianGen, DP-424) according to the manufacturer’s instructions. Typically, 1 μg of total RNA was reversely transcribed to cDNA using a first-strand cDNA synthesis kit (Fermantas, K1632). Gene expressions were quantified using LightCycler480 SYBR Green I Master (Roche, 4707516001) on a LightCycler480 system (Roche). All reactions were run in replicates of at least three samples. The following primers were used: mouse *Actin*: forward, 5′-GCCAACCGTGAAAAGATGAC-3′ and reverse, 5′-GAGGCATACAGGGACAGCAC-3′; mouse *RNF220*: forward, 5′-GTCTCAGTAGACAAGGACGTTCACA-3′ and reverse, 5′-GGGGTGGAGGTGTAGTAAGGAAG-3′; mouse *Olig1*: forward, 5′-TCTTCCACCGCATCCCTTCT-3′ and reverse, 5′-CCGAGTAGGGTAGGATAACTTCG-3′; mouse *Olig2*: forward, 5′-TCCCCAGAACCCGATGATCTT-3′ and reverse, 5′-CGTGGACGAGGACACAGTC-3′; mouse *MBP*: forward, 5′-GACCATCCAAGAAGACCCCAC-3′ and reverse, 5′-GCCATAATGGGTAGTTCTCGTGT-3′; mouse *PDGFR*α: forward, 5′-ACACGTTTGAGCTGTCAACC-3′ and reverse, 5′-CCCGACCACACAAGAACAGG-3′; mouse *GFAP*: forward, 5′-CGGAGACGCATCACCTCTG-3′ and reverse, 5′-AGGGAGTGGAGGAGTCATTCG-3′; mouse *PSD95*: forward, 5′-CAAGGATGGCAGGTTGCAGATCGGA-3′ and reverse, 5′-TCCTCATGCATGACATCCTCTAG-3′; mouse *PLP*: forward, 5′-CCAGAATGTATGGTGTTCTCCC-3′ and reverse, 5′-GGCCCATGAGTTTAAGGACG-3′; mouse *MAG*: forward, 5′-CTGCCGCTGTTTTGGATAATGA-3′ and reverse, 5′-CATCGGGGAAGTCGAAACGG-3′; mouse *MOG*: forward, 5′-AGCTGCTTCCTCTCCCTTCTC-3′ and reverse, 5′-ACTAAAGCCCGGATGGGATAC-3′.

### Immunofluorescence analysis and myelin staining

Transverse brain sections of 12-μm thickness were prepared and HEK293 cells were seeded on LAB-TEK chamber slides (Thermo Fisher Scientific, 154453) for immunoflorescence assays as previously described (*18*, *42*). The following antibodies were used: anti-RNF220 (1:200; Sigma-Aldrich, HPA027578), anti-PDGFRα (1:400; R&D, AF1026), anti-CC1 (1:200; Millipore, OP80), anti-Sox10 (1:300; Oasis Biofarm, OB-PGP001), anti-BrdU (1:200; Bio-Rad, MCA6144), anti-Ki67 (1:200; Abcam, ab15580), and fluorescence-conjugated secondary antibodies, Alexa Fluor 488/555/594 donkey/goat anti-rabbit/mouse/rat/ guinea pig immunoglobulin G (1:400; Invitrogen; A11076, A11055, A21202, A48269, A21206, A31572). Images were captured using a light microscope (Olympus, VS120) and analyzed with ImageJsoftware.

For myelination analysis, 20-μm brain coronal sections were prepared with a cryostat microtome (Leica, CM1850UV) and stained with the Black-Gold II kit (Biosensis, TR-100-BG) according to the manufacturers’ instructions. Images were captured using a light microscope (Olympus, VS120) and analyzed with ImageJ software.

### Electron microscopy

Mice were deeply anesthetized, perfused with cold PBS followed by 4% paraformaldehyde (Sigma-Aldrich, 158172). The corpus callosum tissues were dissected on ice and cut into small pieces. Then, pieces of corpus callosum were postfixed with 2.5% glutaraldehyde (Sigma-Aldrich, G5882) overnight at 4°C; treated with 1% osmium tetroxide (Sigma-Aldrich, 1.24505), ethanol (Sigma-Aldrich, 459828) dehydrated, and acetone (Sigma-Aldrich, 270725) transition; and embedded into epoxy resins (Structure Probe, SPI-Pon 812). Ultrathin sections (60 nm) were stained with 2% uranyl acetate (Electron Microscopy Sciences, 22400-2) and lead citrate (Electron Microscopy Sciences, 22410) for electron microscopy imaging using JEM-1400 plus (JEOL). The extent of axonal myelination was quantified by calculating the *g*-ratio (the ratio between the inner and the outer diameter of the myelin sheath). ImageJ software was used for measurement of the axonal caliber and axonal counting.

### MRI data acquisition and analysis

MRI data were obtained on a 7.0-T Bruker MR system (BioSpec 70/20 USR, Bruker). The mice were anaesthetized by inhalation of 3% isoflurane (RWD, R510-22-10) before scanning, and physiological parameters were monitored and kept constant during the experiment. Tooth and ear bars were used to restrain the mice for imaging. A two-dimensional rapid acquisition with relaxation enhancement sequence was applied to obtain T2-weighted images with the following parameters: repetition time, 3500 ms; echo time, 45 ms; slice width, 0.6 mm.

### WB, co-IP, and ubiquitination assays

WB, in vitro and in vivo ubiquitination, and co-IP assays were carried out as previously described (*18*, *33*, *41*). The following primary antibodies were used for immunoblotting: anti-Flag (1:5000; Sigma-Aldrich, F7425), anti-Myc (1:5000; Proteintech, 16286-1-AP), anti-hemagglutinin (1:5000; Sigma-Aldrich, H3663), anti-RNF220 (1:1000; Sigma-Aldrich, HPA027578), anti-MBP (1:1000; Sigma-Aldrich, ab9348), anti-PDGFRα (1:1000; BD Biosciences, 558774), anti–glial fibrillary acidic protein (GFAP) (1:1000; Proteintech, 60190-1-Ig), anti-PLP (1:1000; Cell Signaling Technology, 85971S), anti-MOG (1:1000; Proteintech, 12690-1-AP), anti-MAG (1:1000; Proteintech, 14386-1-AP), anti-Olig1 (1:1000; Santa Cruz Biotechnology, SC-166257), anti-Olig2 (1:1000; Millipore, MABN50), anti-ubiquitin (1:1000; Santa Cruz Biotechnology, SC-8017), and anti–α-tubulin (1:5000; Proteintech, 66031-1-Ig). Horseradish peroxidase–coupled goat anti-mouse or rabbit antibodies (Pierce, 31430 and 31460) were used as secondary antibodies. Chemiluminescence detection was conducted using a chemiluminescent protein detection kit (Thermo Fisher Scientific, 34580) according to the manufacturer’s instructions. Immunoblot signals were quantified by ImageJ software.

### Cell sorting

Isolation of OLs and astrocytes was performed as described previously (*43*). Mice were deeply anesthetized and then forebrains were dissected and minced. Then, the tissues were incubated with PBS containing papain (Worthington, LS003119) and deoxyribonuclease I (Thermo Fisher Scientific, EN0521) for 30 min at 25°C. After digestion, the tissues were triturated using a dounce homogenizer and then filtered through a 40-μm cell strainer. Single-cell suspensions were incubated with anti-PDGFRα (Miltenyi, 130-101-502), anti-O4 (Miltenyi, 130-096-670), or anti-ASCA2 microbeads (Miltenyi, 130-097-679) at 4°C for 4 to 6 hours. Cells bound to the beads were harvested for total RNA or protein extraction.

### Mouse behavior tests

All the neural behavioral experiments were approved by the Animal Care and Use Committee of Kunming Institute of Zoology, Chinese Academy of Sciences. All behavioral experiments were performed in the light phase (9:00 a.m. to 6:00 p.m.) in a sound proof room with a neutral environment, and individual tests were performed in relatively fixed time. Eight-week-old mice were used for all tests. The mice were first given a 60-min habituation after transferring to the rooms for behavioral tests. The experimenter was blind to the group identity of the tested mice, and the inner surfaces of instruments were cleaned with 75% alcohol after each test. Mice with different genotypes were conducted in random order.

#### Novel object recognition test

The novel object recognition test consisted of three phases: habituation, training, and test. In the habituation phase, the animals were allowed to explore an empty arena for 5 min. Then, 24 hours later, during the training trial, each mouse was individually placed into the arena containing two identical objects (A1 and A2), equidistant from each other, and allowed to explore the objects for 10 min. After 1 hour, during the test phase, one copy of the familiar object (A3) and a novel object (B) was placed in the same location as during the training trial. The exploring time was recorded when the mouse touched the object with the tip of its nose or the front paws in a total time of 5 min. The discrimination index was calculated as the difference in time exploring the novel and the familiar objects, expressed as the percentage ratio of the total time spent exploring both objects.

#### Three-chamber sociability and social novelty test

Sociability and social novelty tests were performed on mice as previously described with minor modifications (*33*). Both strangers used were WT C57BL/6 mice with matched age, body weight, and sex of the mice being tested. The social test apparatus was made of a clear glass box (60 cm by 40 cm by 30 cm) with three equally divided chambers (40 cm by 30 cm each). The chambers were interconnected with 5 cm by 5 cm openings, which could be opened or closed manually. The inverted cylindrical wire cups, which contain the stranger mouse or an object (ball), were 10 cm in height and contained a 10-cm floor with the metal bars spaced 0.8 cm apart. The day before the test, each of the stranger mice was habituated inside the inverted wire cups, and each of the test mice was habituated to the apparatus with two empty wire cups inside the box for 15 min. On the test day, during the habituation phase, an empty wire cup was placed into the left and right chamber, and the tested mouse was placed into the center chamber and allowed to explore for 5 min, with all doors open between chambers. During the sociability test phase, an unfamiliar mouse (S1) was placed inside the inverted wire cup in one of the side chambers, an object (O) was placed inside the inverted wire cup on another side chamber, and the test mouse was introduced to the center chamber with the doors to both side chambers closed. Then, the doors between chambers were lifted simultaneously, and the test mouse was allowed to explore all three chambers for 10 min. During the social novelty test phase, the test mouse was placed in the central chamber with all doors closed between chambers. After a novel mouse (S2) was introduced in the inverted wired cup, replacing the object (O) in one of the side chambers, the doors between chambers were lifted simultaneously, and the test mouse was allowed to explore all three chambers for an additional 10 min. Time spent in close proximity to the empty cup (E1 and E2) or the stranger mice (S1 and S2) or object (O) was analyzed.

#### Morris water maze test

To assess spatial learning and memory, the Morris water maze test was performed as described previously (*44*). Briefly, a blue circular pool of 120 cm in diameter and was divided into four equal quadrants with two hypothetical crossed lines. The hidden circular platform located in the middle of the target quadrant was 10 cm in diameter and submerged 1 cm below the water surface. During the training period, mice were trained to find the hidden platform over eight consecutive days with three trials everyday using a semirandom set of start locations, with the restriction that only one trial each day started from each of the three different starting positions. If a mouse failed to find the platform within 60 s, it was picked up and placed on the platform for 20 s. On the ninth day, 24 hours after the last training session, the platform was removed, and 60 s was given to each mouse to search for the platform in the pool, with the starting location opposite to the previous position. The movement of the mice was monitored using Noldus software (EthoVision XT 8.0, Noldus Technology). Escape latency to find the platform, total distance moved, average velocity, total distance to platform, and duration in platform zone were automatically analyzed by the software.

#### Contextual fear conditioning test

The contextual fear conditioning test was conducted as previously described with minor modifications (*44*). Mice were placed in a box and received five foot shocks (1.2 mA, 2 s) with 2-min intershock intervals by FreezeFrame (Coulbourn Instruments). Freezing behavior was measured by the amount of time mice exhibited freezing behavior during each intershock interval. Then, mice were placed back in the box (fear context) 30 min, 1 day, 3 days, and 6 days after fear conditioning, and their contextual freezing behavior was measured for 11 min without any foot shocks applied.

#### Rotarod test

Motor coordination was assessed with a rotarod apparatus (Economex, Columbus Instruments) as described previously (*45*). Briefly, for each mouse, testing was done at least three times and the intratrial interval was about 45 to 60 min. The latency (time and speed) to fall of each mouse was recorded, and the results from three trials were averaged as the indicator of motor performance.

### Quantification and statistical analysis

Data analysis was carried out using Excel (Microsoft) and GraphPad Prism (GraphPad Software) and data are presented by mean ± SD. For biochemical analyses and immunostaining assays, all experiments were repeated at least three times independently. Statistical analyses are compared to respective control with Mann-Whitney *U* test with Bonferroni correction. There is no sample or data point excluded or omitted for the analysis reported in this study.
