# Supplementary material for: RNF220-mediated K63-linked polyubiquitination stabilizes Olig proteins during oligodendroglial development and myelination
Source: Sci Adv. 2024 Feb 7;10(6):eadk3931. doi: 10.1126/sciadv.adk3931 (PMC10849602; doi:10.1126/sciadv.adk3931)
Supplement: Supplementary file 1 — Figs. S1 to S14 [file sciadv.adk3931_sm.pdf]

Science Advances
AAAS

Supplementary Materials for

RNF220-mediated K63-linked polyubiquitination stabilizes Olig proteins during oligodendroglial development and myelination

Yuwei Li *et al.*

Corresponding author: Pengcheng Ma, kunmapch@mail.kiz.ac.cn; Nengyin Sheng, shengnengyin@mail.kiz.ac.cn; Bingyu Mao, mao@mail.kiz.ac.cn

Sci. Adv. **10**, eadk3931 (2024)
DOI: 10.1126/sciadv.adk3931

This PDF file includes:

Figs. S1 to S14

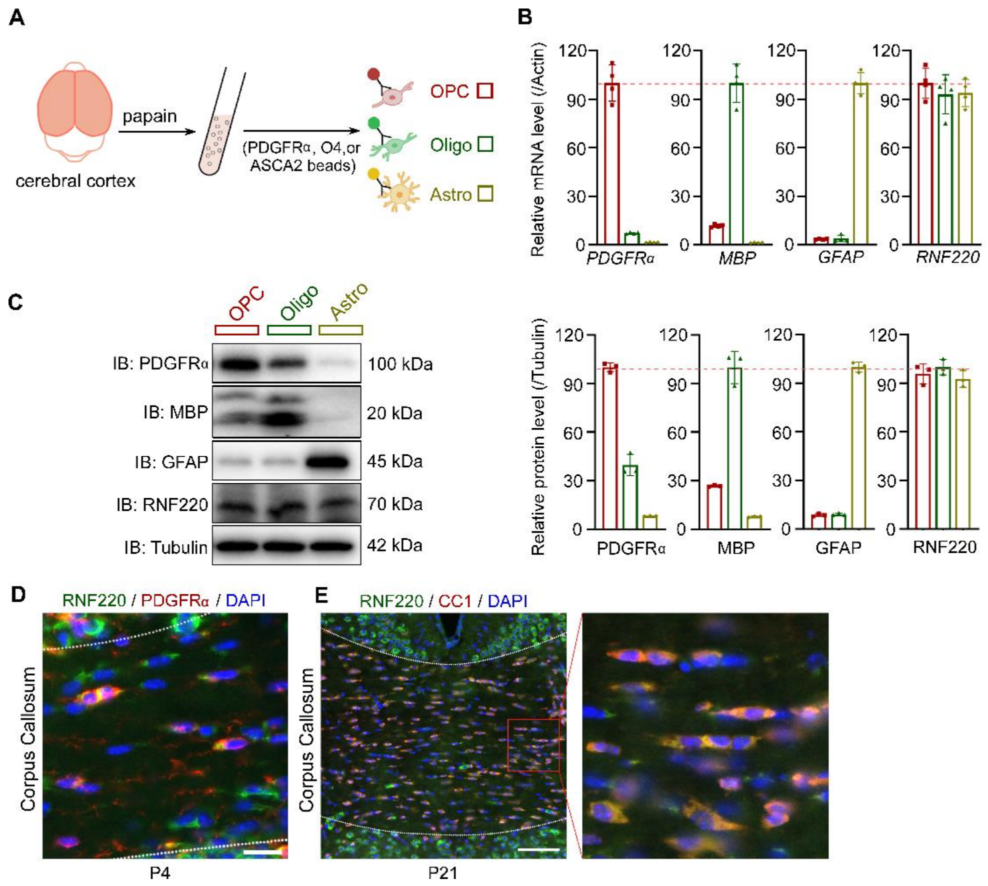

Supplementary Figure 1, related to Figure 1. Expression of RNF220 in OL lineage cells. (**A**) Schematic diagram of isolating astroglial and oligodendroglial cells from mouse forebrains with PDGFRα$^+$, O4$^+$ and ASCA2$^+$ beads. (**B**) Expression of *RNF220* and oligodendroglial genes in the isolated OPCs, OLs and astrocytes, and β-actin was used as the internal control. Bar graphs (mean ± SD) show the relative mRNA levels normalized against indicated genes of the respective cell having highest level (***PDGFRα***: OPC: 100 ± 11.11, OL: 7.24 ± 0.51, astrocyte: 1.43 ± 0.15; ***MBP***: OPC: 11.81 ± 0.93, OL: 100 ± 11.86, astrocyte: 1.27 ± 0.18; ***GFAP***: OPC: 3.51 ± 0.31, OL: 3.81 ± 1.95, astrocyte: 100 ± 6.35; ***RNF220***: OPC: 100 ± 9.39, OL: 92.97 ± 12.14, astrocyte: 93.92 ± 8.40). (**C**) Protein expression of RNF220 and oligodendroglial genes in isolated OPCs, OLs and astrocytes, and β-tubulin was used as the internal control. Bar graphs (mean ± SD) show the relative levels against indicated proteins of the respective cell having highest level (**PDGFRα**: OPC: 100 ± 2.71, OL: 39.70 ± 6.55, astrocyte: 8.22 ± 0.17; **MBP**: OPC: 26.74 ± 0.56, OL: 100 ± 9.96, astrocyte: 7.77 ± 0.33; **GFAP**: OPC: 8.61 ± 0.87, OL: 9.06 ± 0.72, astrocyte: 100 ± 3.01; **RNF220**: OPC: 95.77 ± 6.09, OL: 100 ± 4.78, astrocyte: 92.62 ± 5.22).

IB: immunoblotting. (**D** and **E**) Immunofluorescence staining assays show RNF220 expression in PDGFRα$^+$ OPCs (D) and CC1$^+$ OLs (E) in corpus callosum region of P4 and P21 respectively. Scale bars, 50 μm for (D) and 100 μm for (E).

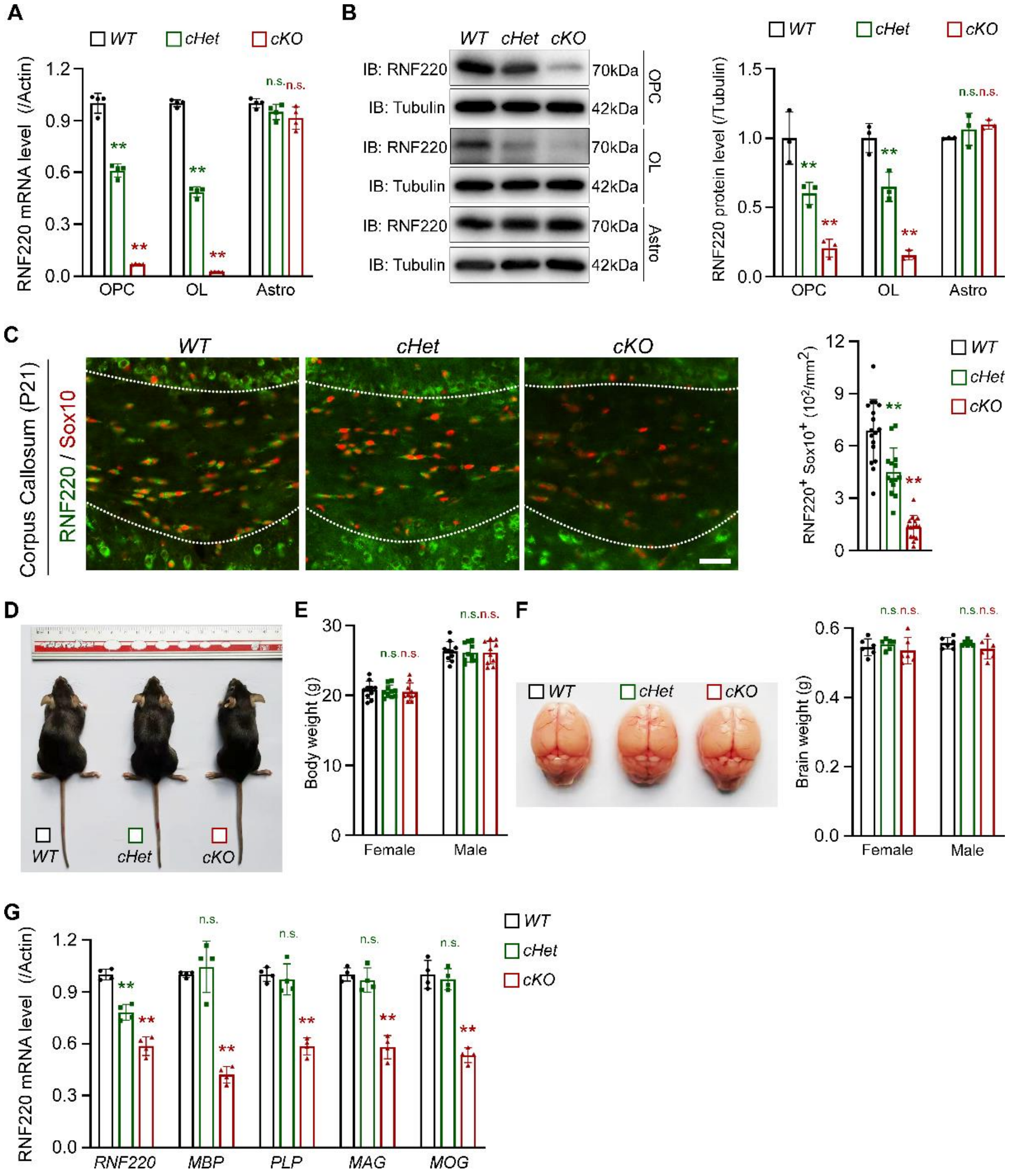

Supplementary Figure 2, related to Figure 1. RNF220 expression and brain morphology of *RNF220-cKO* mice. (**A**) Bar graphs (mean ± SD) show the relative mRNA levels of *RNF220* in isolated OPCs, OLs and astrocytes normalized against expression in the respective wild-type controls (**OPC**: *WT*: 1.00 ± 0.06, *cHet*: 0.61 ± 0.37, *cKO*: 0.07 ± 0.00; **OL**: *WT*: 1.00 ± 0.02, *cHet*: 0.49 ± 0.03, *cKO*: 0.03 ± 0.00; **astrocyte**: *WT*: 1.00 ± 0.03, *cHet*: 0.94 ± 0.04, *cKO*: 0.91 ± 0.06). (**B**) Protein levels of RNF220 in isolated OPCs, OLs and astrocytes, and β-tubulin was used as the internal control. Bar graphs (mean ± SD) show the relative levels against RNF220 expression in the respective wild-type controls (**OPC**: *WT*: 1.00 ± 0.19, *cHet*: 0.60 ± 0.08, *cKO*: 0.21 ± 0.06; **OL**: *WT*: 1.00 ± 0.10, *cHet*: 0.64 ± 0.10, *cKO*: 0.15 ± 0.03;

astrocyte: *WT*: 1.00 ± 0.00, *cHet*: 1.06 ± 0.12, *cKO*: 1.10 ± 0.03). IB: immunoblotting. (**C**) Immunofluorescence staining showing the expression of RNF220 in the corpus callosum Sox10$^+$ OPCs of P21 *RNF220-WT*, *RNF220-cHet* and *RNF220-cKO* mice. Scale bars, 40 μm. Bar graphs (mean ± SD) show quantification of RNF220$^+$Sox10$^+$ cells (*WT*: 6.90 ± 1.78×10^2 / mm^2; *cHet*: 4.52 ± 1.38×10^2 / mm^2; *cKO*: 1.34 ± 0.66×10^2 / mm^2). (**D**) Representative gross view of P60 *RNF220-WT*, *RNF220-cHet* and *RNF220-cKO* mice. (**E**) Bar graphs (mean ± SD) show body weight of the male (n = 10 / genotype) and female (n = 10 / genotype) mice of indicated genotypes (**Female**: *WT*: 20.78 ± 1.25 g, *cHet*: 20.6 ± 0.88 g, *cKO*: 20.5 ± 1.27 g; **Male**: *WT*: 26.29 ± 1.41 g, *cHet*: 26.11 ± 1.31 g, *cKO*: 26.11 ± 1.63 g). (**F**) Representative brain morphology of P60 *RNF220-WT*, *RNF220-cHet* and *RNF220-cKO* mice. Bar graphs (mean ± SD) show brain weight of the male (n = 6 / genotype) and female (n = 6 / genotype) mice of indicated genotypes (**Female**: *WT*: 0.55 ± 0.02 g, *cHet*: 0.55 ± 0.02 g, *cKO*: 0.54 ± 0.04 g; **Male**: *WT*: 0.56 ± 0.02 g, *cHet*: 0.56 ± 0.01 g, *cKO*: 0.54 ± 0.03 g). (**G**) Realtime-PCR analyses of mRNA expression of *RNF220*, *MBP*, *PLP*, *MAG*, and *MOG* in the cerebral cortex of P21 *RNF220-WT* (n = 4), *RNF220-cHet* (n = 4), and *RNF220-cKO* (n = 4) mice, and β-actin was used as the internal controls. Bar graphs (mean ± SD) show the relative mRNA levels normalized against indicated genes expression in the respective wild-type controls (***RNF220***: *WT*: 1.00 ± 0.03, *cHet*: 0.78 ± 0.05, *cKO*: 0.59 ± 0.05; ***MBP***: *WT*: 1.00 ± 0.02, *cHet*: 1.01 ± 0.15, *cKO*, 0.42 ± 0.05; ***PLP***: *WT*: 1.00 ± 0.04, *cHet*: 0.97 ± 0.09, *cKO*: 0.58 ± 0.05; ***MAG***: *WT*: 1.00 ± 0.04, *cHet*: 0.97 ± 0.07, *cKO*: 0.58 ± 0.07; ***MOG***: *WT*: 1.00 ± 0.08, *cHet*: 0.97 ± 0.06, *cKO*: 0.53 ± 0.04). IB: immunobloting; WT: wild-type. Statistical analyses are compared to respective control with Mann-Whitney U test with Bonferroni correction. n.s. (not significant), $P > 0.05$; **, $P < 0.01$.

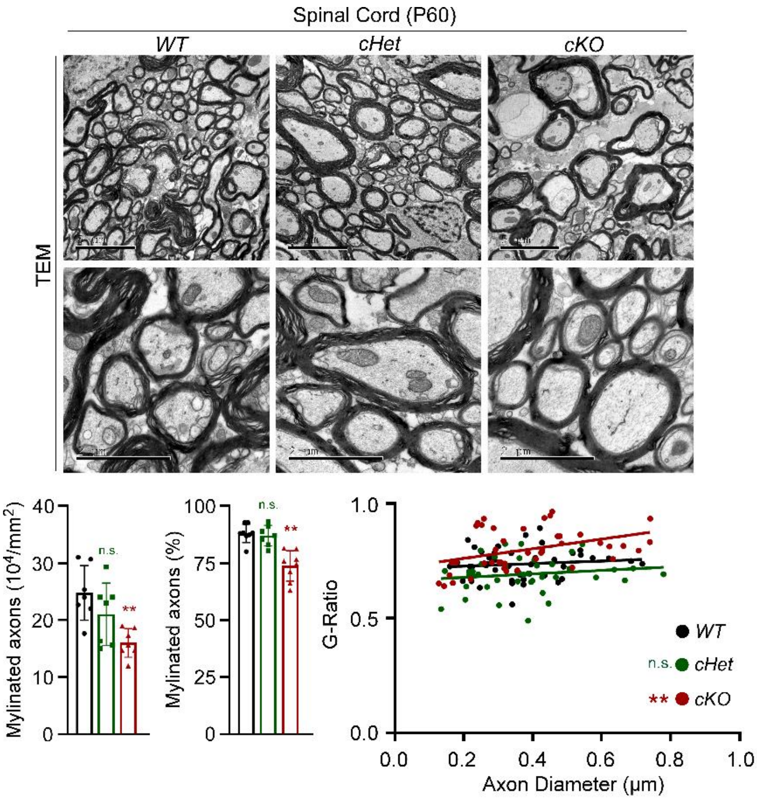

Supplementary Figure 3, related to Figure 1. RNF220 knock-out leads to myelination defects in the spinal cord. Electron microscopy images of the spinal cord transverse sections from P60 *RNF220-WT* (n = 8), *RNF220-cHet* (n = 8), and *RNF220-cKO* (n =8) mice. Scale bar: 5 μm for upper panels and 2 μm for lower panels. Bar graphs (mean ± SD) show quantification of the number (*WT*: 24.77 ± 4.78 × 10^4 / mm^2; *cHet*: 21.05 ± 5.44 × 10^4 / mm^2; *cKO*: 16.00 ± 2.50 × 10^4 / mm^2) and percentage (*WT*: 88.05 ± 3.98%; *cHet*: 87.02 ± 4.51%; *cKO*: 73.76 ± 6.63%) of myelinated axons. Scatterplots show g-ratios relative to axon diameter (*WT*: 0.74 ± 0.07; *cHet*: 0.69 ± 0.07; *cKO*: 0.80 ± 0.09). Statistical analyses are compared to respective control with Mann-Whitney U test with Bonferroni correction. n.s. (not significant), $P > 0.05$; **, $P < 0.01$.

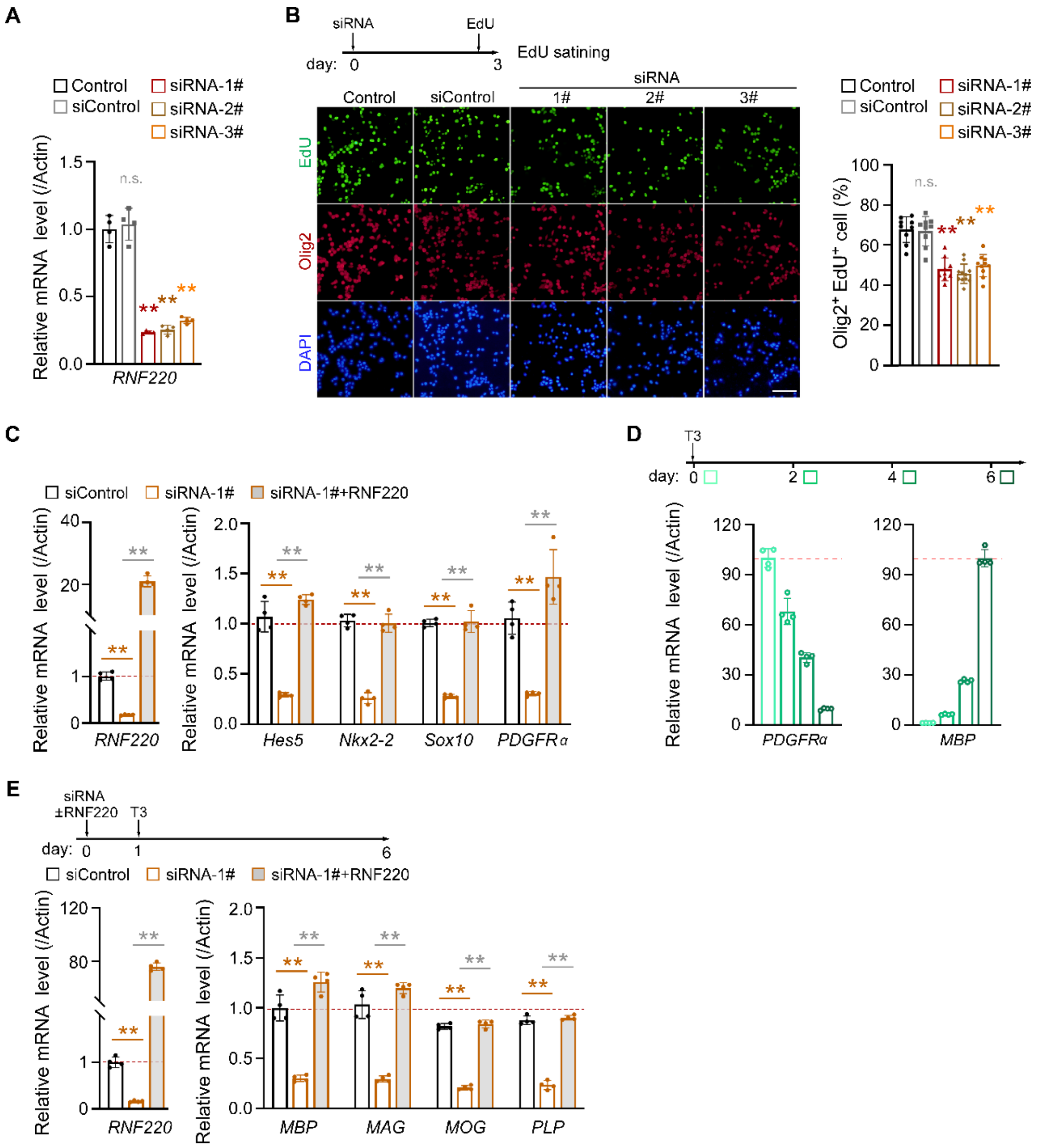

Supplementary Figure 4, related to Figure 2. RNF220 knock-down inhibits proliferation and differentiation of MOPC cells. (**A**) Bar graphs (mean ± SD) show the relative mRNA levels of *RNF220* normalized against expression in the control MOPC cells without siRNA transfection (Control: 1.00 ± 0.10; siControl: 1.04 ± 0.12; siRNA-1#: 0.24 ± 0.01; siRNA-2#: 0.25 ± 0.03; siRNA-3#: 0.32 ± 0.02). (**B**) EdU staining analyses of MOPC cells proliferation after RNF220 knock-down, and bar graphs (mean ± SD) show quantification of Olig2$^+$EdU$^+$ cells (Control: 67.75 ± 6.45%; siControl: 66.98 ± 07.30%; siRNA-1#: 47.9 ± 5.75%; siRNA-2#: 45.72 ± 4.82%; siRNA-3#: 49.73 ± 5.53%). (**C**) RNF220 overexpression restored the down-regulated expression of OPC marker genes in RNF220 knockdown MOPC cells in proliferating.

Bar graphs (mean ± SD) show the relative mRNA expression of *RNF220*, *Hes5*, *Nkx2-2*, *Sox10*, and *PDGFRα* normalized against indicated genes expression in control cells without siRNA transfection in proliferating MOPC cells (***RNF220***: Control: 1.01 ± 0.09, siRNA: 0.18 ± 0.01, siRNA + RNF220: 20.95 ± 1.78; ***Hes5***: Control: 1.07 ± 0.15, siRNA: 0.29 ± 0.02, siRNA + RNF220: 1.24 ± 0.05; ***Nkx2-2***: Control: 1.03 ± 0.06, siRNA-1#: 0.26 ± 0.05, siRNA-1# + RNF220: 1.01 ± 0.09; ***Sox10***: Control: 1.01 ± 0.04, siRNA-1#: 0.28 ± 0.02, siRNA-1# + RNF220: 1.02 ± 0.11; ***PDGFRα***: Control:1.05 ± 0.16, siRNA-1#: 0.30 ± 0.02, siRNA-1# + RNF220: 1.47 ± 0.27). (**D**) Differentiation analyses of MOPC cells with T3 supplements, and bar graphs (mean ± SD) show the relative mRNA levels of *PDGFRα* and *MBP* on indicated days normalized against indicated genes expression in the control cells without T3 treatment (***PDGFRα***: day 0: 100 ± 5.68, day 2: 67.99 ± 7.89, day 4: 40.44 ± 2.94, day 6: 9.54 ± 0.65; ***MBP***: day 0: 1.30 ± 0.09, day 2: 6.46 ± 0.33, day 4: 26.47 ± 0.98, day 6: 100 ± 5.17). (**E**) RNF220 overexpression restored the down-regulated expression of OL marker genes in RNF220 knockdown MOPC cells in differentiating state. Bar graphs (mean ± SD) show the relative mRNA expression of *RNF220*, *MBP*, *MAG*, *MOG*, and *PLP* normalized against indicated genes expression in control cells without siRNA transfection in differentiating MOPC cells (***RNF220***: Control: 1.00 ± 0.11, siRNA-1#: 0.15 ± 0.02, siRNA-1# + RNF220: 75.6 ± 2.94; ***MBP***: Control: 1.00 ± 0.13, siRNA-1#: 0.30 ± 0.03, siRNA-1# + RNF220: 1.26 ± 0.10; ***MAG***: Control: 1.04 ± 0.14, siRNA-1#: 0.29 ± 0.03, siRNA-1# + RNF220: 1.20 ± 0.05; ***MOG***: Control: 0.818 ± 0.03, siRNA-1#: 0.21 ± 0.02, siRNA-1# + RNF220: 0.84 ± 0.04; ***PLP***: Control: 0.88 ± 0.04, siRNA-1#: 0.23 ± 0.04, siRNA-1# + RNF220: 0.90 ± 0.02). Statistical analyses are compared to respective control with Mann-Whitney U test with Bonferroni correction. n.s. (not significant), $P > 0.05$; **, $P < 0.01$.

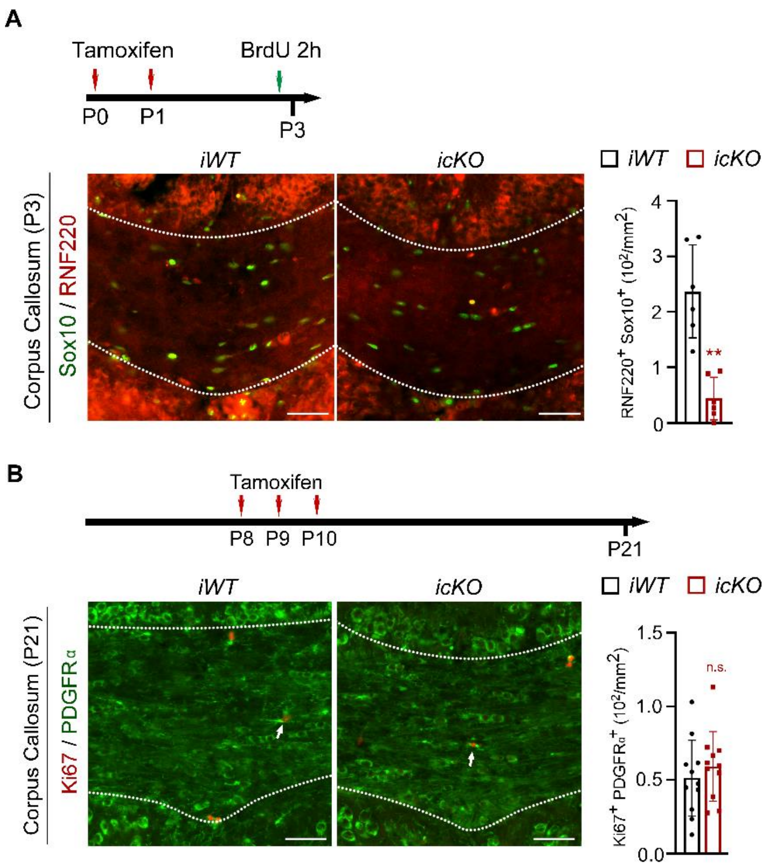

Supplementary Figure 5, related to Figure 2. Tamoxifen induced RNF220 knockout in the corpus callosum of *RNF220-icKO* mice. (**A**) Schematic diagram of tamoxifen induced knockout and BrdU labeling in *RNF220-iWT* and *RNF220-icKO* mice. Immunofluorescence staining of RNF220 and Sox10 in the corpus callosum of *RNF220-iWT* (n = 3) and *RNF220-icKO* (n =3) on P3, and bar graphs (mean ± SD) show the quantification of RNF220$^+$Sox10$^+$ cells (*iWT*: 2.37 ± 0.84 × 10^2 / mm^2; *icKO*: 0.44 ± 0.39 × 10^2 / mm^2). Scale bars, 50 μm. (**B**) Schematic diagram of tamoxifen induced knockout in *RNF220-iWT* and *RNF220-icKO* mice. Immunofluorescence staining of Ki67 and PDGFRα in the corpus callosum of *RNF220-iWT* (n = 3) and *RNF220-icKO* (n =3) on P21, and bar graphs (mean ± SD) show the quantification of Ki67$^+$PDGFRα$^+$ cells (*iWT*: 0.51 ± 0.26 × 10^2 / mm^2; *icKO*: 0.59 ± 0.24 × 10^2 / mm^2). White arrows show the cells quantified with both high Ki67 and PDGFRα signals. Scale bars, 50 μm. Statistical analyses are compared to respective control with Mann-Whitney U test with Bonferroni correction. *, $P < 0.01$; **, $P < 0.01$.

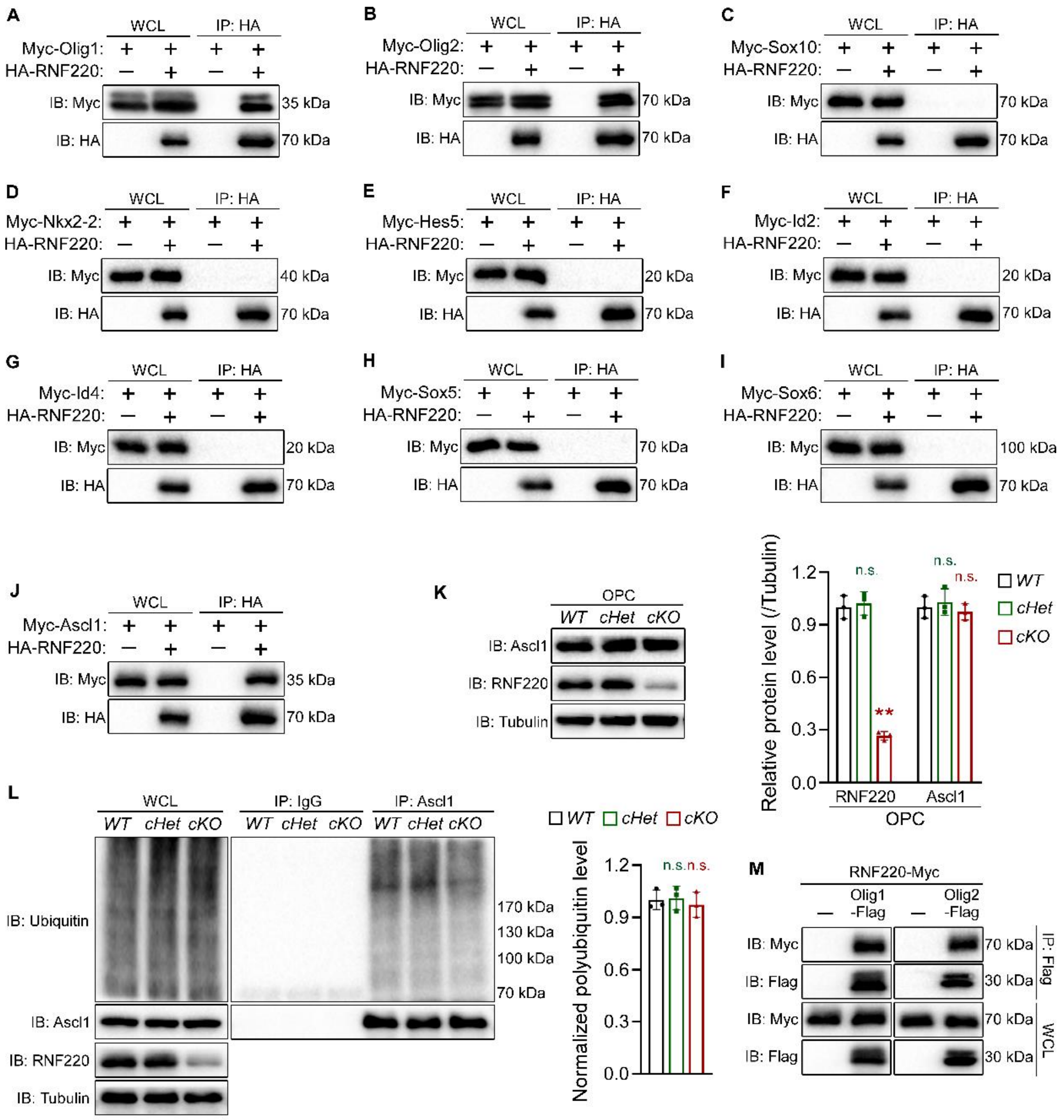

Supplementary Figure 6, related to Figure 5. The interaction between RNF220 and master transcriptional factors for oligodendroglial development. (**A** to **J**) Using HA-RNF220 as a bait, co-IP analyses of the interaction between RNF220 and the indicated transcription factors, including Olig1, Olig2, Sox10, Nkx2-2, Hes5, Id2, Id4, Sox5, Sox6, and Ascl1, when overexpressed in HEK293 cells. (**K**) Western blots analyses of endogenous protein levels of Ascl1 and RNF220 in OPC cells isolated from P7 brains of *RNF220-WT* (n = 3), *RNF220-cHet* (n = 3), or *RNF220-cKO* (n = 3) mice, and β-tubulin was used as the internal control. Bar graphs (mean ± SD) show the relative levels normalized against indicated proteins expression in the respective wild-type controls (**RNF220**: *WT*: 1.00 ± 0.07, *cHet*: 1.02 ± 0.07, *cKO*: 0.26 ± 0.02; **Ascl1**: *WT*: 1.00 ± 0.06, *cHet*: 1.03 ± 0.08, *cKO*: 0.97 ± 0.05). (**L**) Western blots

analyses of Ascl1 polyubiquitination in the forebrains of P7 *RNF220-WT* (n = 3) and *RNF220-cKO* (n = 3) mice, and bar graphs (mean ± SD) show normalized polyubiquitination levels against expression in the wild-type controls (*WT*: 1.00 ± 0.06; *cHet*: 1.01 ± 0.07; *cKO*: 0.97 ± 0.07). (**M**) Using Flag-Olig1 or Flag-Olig2 as a bait, co-IP analyses of the interaction between RNF220 and Olig1 or Olig2 in HEK293 cells. IB: immunoblotting; IP: immunoprecipitation; WCL: whole cell lysate; WT: wild-type. Statistical analyses are compared to respective control with Mann-Whitney U test with Bonferroni correction. n.s. (not significant), $P > 0.05$; **, $P < 0.01$.

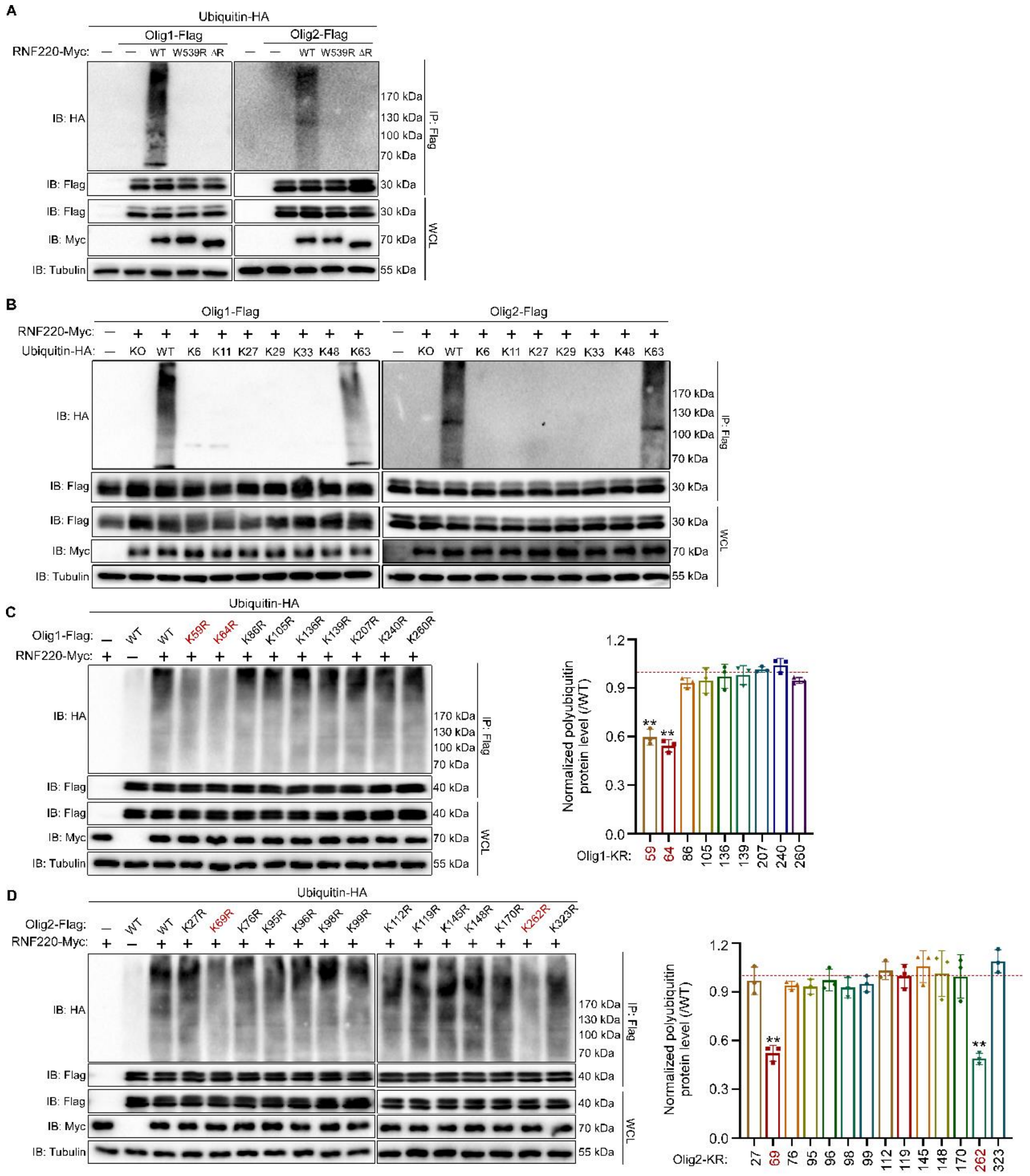

Supplementary Figure 7, related to Figure 5. RNF220 targets Olig1 and Olig2 for K63-linked polyubiquitination. (**A**) Ubiquitination analyses of polyubiquitinated Olig1 or Olig2 when co-expressed with wild-type RNF220 or E3 ligase-dead mutants, RNF220^{W539R} and RNF220$^{\Delta RING}$, in HEK293 cells. (**B**) Ubiquitination analyses of RNF220-mediated polyubiquitination of Olig1 or Olig2 when co-expressed with the indicated ubiquitin mutants in HEK 293 cells. (**C**) Ubiquitination analyses of RNF220-mediated polyubiquitination of Olig1 mutants with the indicated lysine mutated into arginine in HEK 293 cells, and bar graphs (mean ± SD) show normalized polyubiquitination levels against expression of the wild-type control (K59R: 0.60 ±

0.05; K64R: 0.54 ± 0.04; K86R: 0.93 ± 0.03; K105R: 0.95 ± 0.08; K136R: 0.97 ± 0.07; K139R: 0.98 ± 0.06; K207R: 1.02 ± 0.02; K240R: 1.04 ± 0.04; K260R: 0.95 ± 0.02). (**D**) Ubiquitination analyses of RNF220-mediated polyubiquitination of Olig2 mutants with the indicated lysine mutated into arginine in HEK 293 cells, and bar graphs (mean ± SD) show normalized polyubiquitination levels against expression of the wild-type control (K27R: 0.97 ± 0.08; K69R: 0.52 ± 0.05; K76R: 0.94 ± 0.05; K96R: 0.97 ± 0.07; K98R: 0.93 ± 0.06; K99R: 0.95 ± 0.05; K112R: 1.03 ± 0.06; K119R: 1 ± 0.07; K145R: 1.05 ± 0.10; K148R: 1.01 ± 0.14; K170R: 0.99 ± 0.13; K262R: 0.49 ± 0.03; K323R: 1.09 ± 0.07). IB: immunoblotting; IP: immunoprecipitation; WCL: whole cell lysate; WT: wild-type; ΔR: ΔRING. Statistical analyses are compared to respective control with Mann-Whitney U test with Bonferroni correction. n.s. (not significant), $P > 0.05$; **, $P < 0.01$.

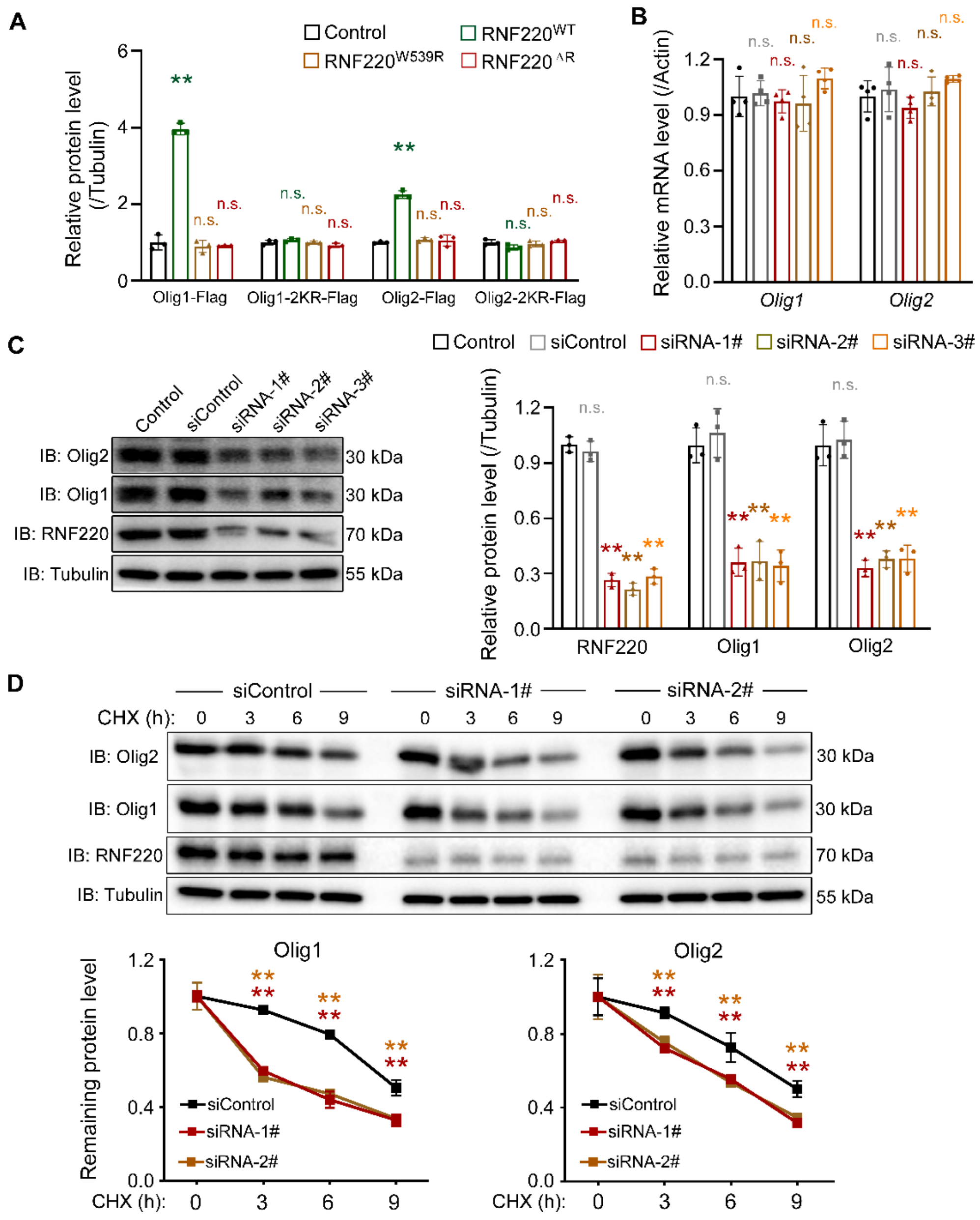

Supplementary Figure 8, related to Figure 6. RNF220 knock-down downregulates Olig1 and Olig2 protein expression in MOPC cells. (**A**) Western blot analyses of RNF220-regulated protein expression of Olig1/ Olig1^{2KR} and Olig2/ Olig2^{2KR} in HEK293 cells, and bar graphs (mean ± SD) show the relative levels of Olig1 and Olig2 normalized against expression in the respective controls without RNF220 co-transfection (**Olig1**: Control: 1.00 ± 0.20, RNF220^{WT}: 3.96 ± 0.15, RNF220^{W539R}: 0.90 ± 0.16, RNF220$^{\Delta RING}$: 0.91 ± 0.01; **Olig1^{2KR}**: Control: 1.00 ± 0.06, RNF220^{WT}: 1.06 ± 0.04, RNF220 ^{W539R}: 1.00 ± 0.04, RNF220$^{\Delta RING}$: 0.93 ± 0.05; **Olig2**: Control: 1.00 ± 0.20, RNF220^{WT}: 2.25 ± 0.10, RNF220^{W539R}: 1.07 ± 0.05, RNF220$^{\Delta RING}$: 1.05 ± 0.15; **Olig2^{2KR}**: Control: 1.00 ± 0.07, RNF220^{WT}: 0.87 ± 0.07,

RNF220^{W539R}: 0.96 ± 0.07, RNF220$^{\Delta RING}$: 1.04 ± 0.02). (**B**) Bar graphs (mean ± SD) show the relative mRNA levels of *Olig1* and *Olig2* normalized against respective expression in the control MOPC cells without siRNA transfection (***Olig1***: Control: 1.00 ± 0.13, siControl: 1.07 ± 0.15, siRNA-1#: 0.94 ± 0.09, siRNFA-2#: 1.05 ± 0.07, siRNA-3#: 1.08 ± 0.16; ***Olig2***: Control: 1.00 ± 0.09, siControl: 1.07 ± 0.16, siRNA-1#: 1.13 ± 0.05, siRNA-2#: 1.01 ± 0.12, siRNA-3#: 1.06 ± 0.16). (**C**) Western blots analyses of endogenous protein expression of RNF220, Olig1 and Olig2 in MOPC cells knocking-down RNF220 by siRNAs, and bar graphs (mean ± SD) show the relative levels of indicated proteins normalized against respective expression in the control MOPC cells without siRNA transfection (**RNF220**: Control: 1.00 ± 0.04, siControl: 0.96 ± 0.06, siRNA-1#: 0.26 ± 0.04, siRNA-2#: 0.22 ± 0.03, siRNA-3#: 0.29 ± 0.04; **Olig1**: Control: 1.00 ± 0.10, siControl: 1.07 ± 0.13, siRNA-1#: 0.36 ± 0.08, siRNA-2#: 0.37 ± 0.11, siRNA-3#: 0.34 ± 0.09; **Olig2**: Control: 1.00 ± 0.11, siControl: 1.03 ± 0.10, siRNA-1#: 0.33 ± 0.05, siRNA-2#: 0.38 ± 0.05, siRNA-3#: 0.38 ± 0.07). (**D**) Cycloheximide chase analyses of protein half-lives of endogenous Olig1 and Olig2 in MOPC cells knocking-down RNF220 by siRNAs, and broken line graph (mean ± SD) show normalized levels against indicated proteins expression from the respective control without cycloheximide treatment (**Olig1**: Control: 0 h: 1.00 ± 0.02, 3 h: 0.93 ± 0.02, 6 h: 0.79 ± 0.02, 9 h: 0.50 ± 0.04; siRNA-1#: 0 h: 1.00 ± 0.03, 3 h: 0.60 ± 0.01, 6 h: 0.44 ± 0.04, 9 h: 0.33 ± 0.03; siRNA-2#: 0 h: 1.00 ± 0.07, 3 h: 0.56 ± 0.02, 6 h: 0.47 ± 0.02, 9 h: 0.34 ± 0.02; **Olig2**: Control: 0 h: 1.00 ± 0.01, 3 h: 0.91 ± 0.03, 6 h: 0.73 ± 0.08, 9 h: 0.50 ± 0.04; siRNA-1#: 0 h: 1.00 ± 0.02, 3 h: 0.72 ± 0.02, 6 h: 0.55 ± 0.02, 9 h: 0.32 ± 0.02; siRNA-2#: 0 h: 1.00 ± 0.12, 3 h: 0.75 ± 0.03, 6 h: 0.53 ± 0.01, 9 h, 0.35 ± 0.01). IB: immunoblotting; IP: immunoprecipitation; WCL: whole cell lysate; WT: wild-type; ΔR: ΔRING; CHX: cycloheximide. Statistical analyses are compared to respective control with Mann-Whitney U test with Bonferroni correction. n.s. (not significant), $P > 0.05$; **, $P < 0.01$.

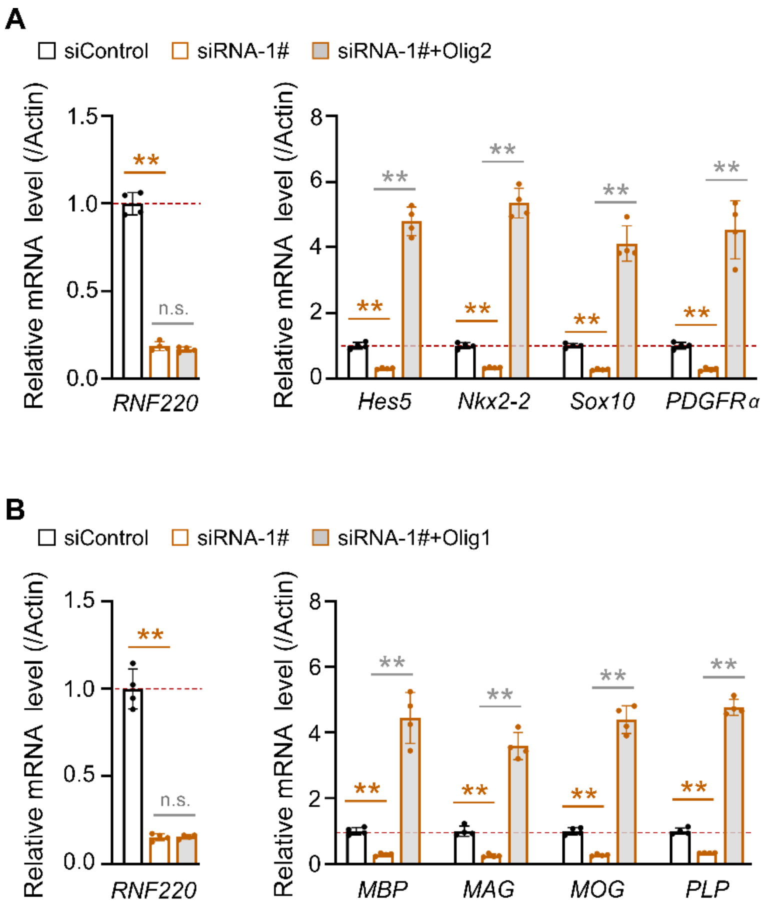

Supplementary Figure 9, related to Figure 6. RNF220 siRNA down-regulated expression of OPC or OL maker genes were restored by Olig1 or Olig2 overexpression in MOPC cells. Olig2 or Olig1 overexpression reversed the down-regulated expression of OPC or OL marker genes in RNF220 knockdown MOPC cells in proliferating or differentiating state respectively. (**A**) Bar graphs (mean ± SD) show the relative mRNA expression of *RNF220*, *Hes5*, *Nkx2-2*, *Sox10*, and *PDGFRα* normalized against indicated genes expression in control cells without siRNA transfection in proliferating MOPC cells ***(RNF220***: Control: 1.00 ± 0.06, siRNA-1#: 0.19 ± 0.03, siRNA-1# + RNF220: 0.17 ± 0.01; ***Hes5***: Control: 1.00 ± 0.10, siRNA-1#: 0.30 ± 0.02, siRNA-1# + RNF220: 4.80 ± 0.44; ***Nkx2-2***: Control: 1.00 ± 0.09, siRNA-1#: 0.33 ± 0.02, siRNA-1# + RNF220: 5.35 ± 0.45; ***Sox10***: Control: 1.00 ± 0.07, siRNA-1#: 0.27 ± 0.03, siRNA-1# + RNF220 4.12 ± 0.54; ***PDGFRα***: Control: 1.00 ± 0.10, siRNA-1#: 0.28 ± 0.05, siRNA-1# + RNF220: 4.53 ± 0.88). (**B**) Bar graphs (mean ± SD) show the relative mRNA expression of *RNF220*, *MBP*, *MAG*, *MOG*, and *PLP* normalized against indicated genes expression in control

cells without siRNA transfection in differentiating MOPC cells (***RNF220***: Control: 1.00 ± 0.11, siRNA-1#: 0.15 ± 0.02, siRNA-1# + RNF220: 0.16 ± 0.01; ***MBP***: Control: 1.00 ± 0.11, siRNA-1#: 0.29 ± 0.04, siRNA-1# + RNF220: 4.45 ± 0.78; ***MAG***: Control: 1.00 ± 0.16, siRNA-1#: 0.25 ± 0.05, siRNA-1# + RNF220: 3.59 ± 0.42; ***MOG***: Control: 1.00 ± 0.11, siRNA-1#: 0.27 ± 0.03, siRNA-1# + RNF220: 4.40 ± 0.42; ***PLP***: Control: 1.00 ± 0.10, siRNA-1#: 0.33 ± 0.01, siRNA-1# + RNF220: 4.78 ± 0.25). Statistical analyses are compared to respective control with Mann-Whitney U test with Bonferroni correction. **, $P < 0.01$.

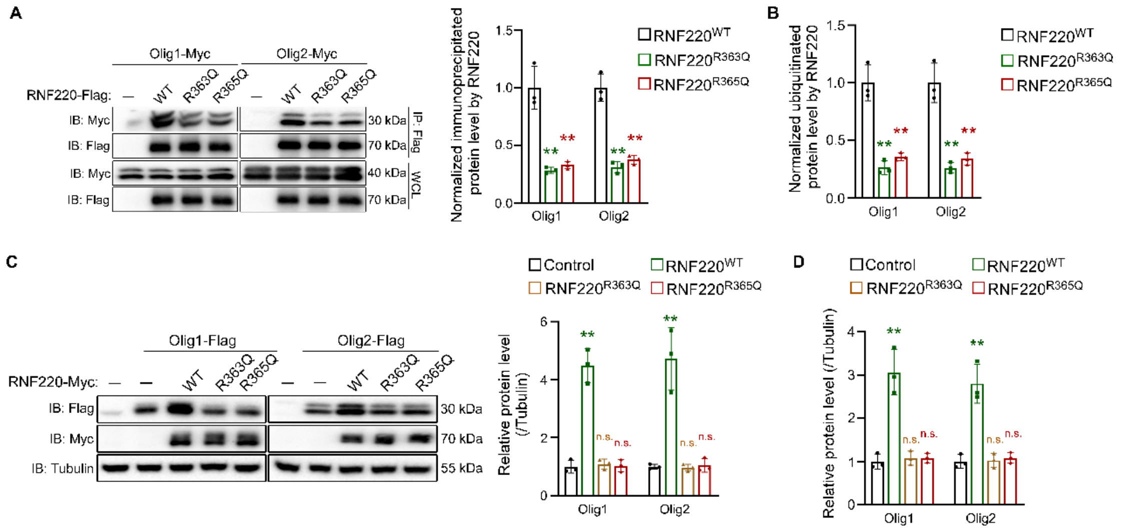

Supplementary Figure 10, related to Figure 7. The effects of leukodystrophy-related RNF220 mutations on Olig1 and Olig2 stabilization. (**A**) Using Flag-RNF220 as a bait, co-IP analyses of the binding of RNF220^{R363Q} and RNF220^{R365Q} mutants with Olig proteins in HEK293 cells, and bar graphs (mean ± SD) show normalized immunoprecipitated protein levels against respective wild-type controls (**Olig1**: RNF220^{WT}: 1.00 ± 0.19, RNF220^{R363Q}: 0.29 ± 0.03, RNF220^{R365Q}: 0.33 ± 0.37; **Olig2**: RNF220^{WT}: 1.00 ± 0.12, RNF220^{R363Q}: 0.31 ± 0.05, RNF220^{R365Q}: 0.37 ± 0.04). (**B**) Ubiquitination analyses of polyubiquitinated Olig1 or Olig2 when co-expressed with wild-type RNF220, RNF220^{R363Q} or RNF220^{R365Q} in HEK293 cells, and bar graphs (mean ± SD) show normalized polyubiquitination levels against expression of the wild-type control (**Olig1**: RNF220^{WT}: 1.00 ± 0.16, RNF220^{R363Q}: 0.26 ± 0.06, RNF220^{R365Q}: 0.36 ± 0.03; **Olig2**: RNF220^{WT}: 1.00 ± 0.17, RNF220^{R363Q}: 0.26 ± 0.05, RNF220^{R365Q}: 0.34 ± 0.05). (**C**) Western blot analyses of protein expression of Olig1 or Olig2 when co-expressed with wild-type RNF220 or RNF220^{R363Q} and RNF220^{R365Q} mutants in HEK293 cells, and bar graphs (mean ± SD) show the relative levels of Olig1 and Olig2 normalized against expression in the respective controls without RNF220 co-transfection (**Olig1**: Control: 1.00 ± 0.22, RNF220^{WT}: 4.47 ± 0.59, RNF220^{R363Q}: 1.09 ± 0.17, RNF220^{R365Q}: 1.03 ± 0.21; **Olig2**: Control: 1.00 ± 0.08, RNF220^{WT}: 4.71 ± 1.08, RNF220^{R363Q}: 0.97 ± 0.12, RNF220^{R365Q}: 1.04 ± 0.23). (**D**) Western blot analyses of endogenous Olig1 and Olig2 expression in MOPC cells overexpressing wild-type RNF220, RNF220^{R363Q} or

RNF220^{R365Q}, and bar graphs (mean ± SD) show the relative levels of indicated proteins normalized against respective expression in the control MOPC cells without transfection (**Olig1**: Control: 1.00 ± 0.17, RNF220^{WT}: 3.01 ± 0.53, RNF220^{R363Q}: 1.08 ± 0.16, RNF220^{R365Q}: 1.08 ± 0.11; **Olig2**: Control: 1.00 ± 0.16, RNF220^{WT}: 2.79 ± 0.45, RNF220^{R363Q}: 1.03 ± 0.17, RNF220^{R365Q}: 1.08 ± 0.13). IB: immunobloting; IP: immunoprecipitation; WCL: whole cell lysate; WT: wild-type. Statistical analyses are compared to respective control with Mann-Whitney U test with Bonferroni correction. n.s. (not significant), $P > 0.05$; **, $P < 0.01$.

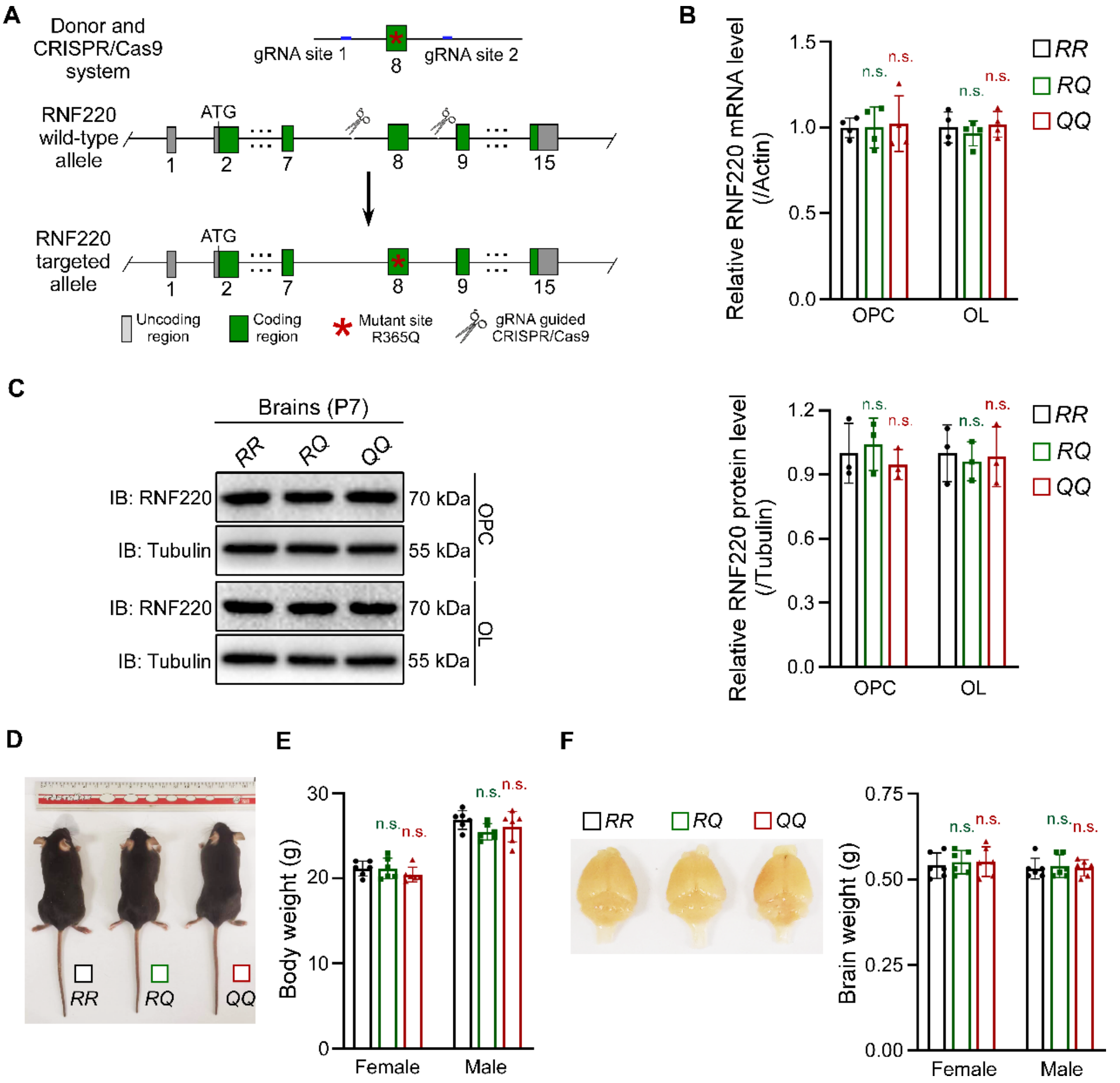

Supplementary Figure 11, related to Figure 7. Generation and brain morphology of RNF220^{R365Q} knock-in mice. (**A**) Strategy for the generation of RNF220^{R365Q} knock-in mice using CRISPR/Cas9 system. (**B**) Bar graphs (mean ± SD) show the relative mRNA levels of *RNF220* in isolated OPC and OL cells from the brains of P7 *RNF220-RR* (n = 4), *RNF220-RQ* (n = 4) and *RNF220-QQ* (n = 4) normalized against its expression in the respective wild-type controls (**OPC**: *RR*: 1.00 ± 0.06, *RQ*: 1.00 ± 0.12, *QQ*: 1.02 ± 0.16; **OL**: *RR*: 1.00 ± 0.09, *RQ*: 0.97 ± 0.07, *QQ*: 1.02 ± 0.07). (**C**) Western blots analyses of RNF220 in isolated OPC and OL cells from the brains of P7 *RNF220-RR* (n = 3), *RNF220-RQ* (n = 3) and *RNF220-QQ* (n = 3) mice, and bar graphs (mean ± SD) show normalized levels against respective its protein expression in the wild-type controls (**OPC**: *RR*: 1.00 ± 0.14, *RQ*: 1.04 ± 0.12, *QQ*: 0.95 ± 0.07; **OL**: *RR*: 1.00 ± 0.13, *RQ*: 0.96 ± 0.09, *QQ*: 0.98 ± 0.14). (**D**) Representative gross

view of P60 *RNF220-RR*, *RNF220-RQ* and *RNF220-QQ* mice. (**E**) Bar graphs (mean ± SD) show body weight of the male (n = 6 / genotype) and female (n = 6 / genotype) mice of indicated genotypes (**Female**: *RR*: 21.17 ± 0.86 g, *RQ*: 21.18 ± 1.23 g, *QQ*: 20.46 ± 0.88 g; **Male**: *RR*: 26.90 ± 1.10 g, *RQ*: 25.49 ± 0.95 g, *QQ*: 26.08 ± 1.80 g). (**F**) Representative brain morphology of P60 *RNF220-RR*, *RNF220-RQ* and *RNF220-QQ* mice. Bar graphs (mean ± SD) show brain weight of the male (n = 6 / genotype) and female (n = 6 / genotype) mice of indicated genotypes (**Female**: *RR*: 0.54 ± 0.04 g, *RQ*: 0.55 ± 0.03 g, *QQ*: 0.55 ± 0.04 g; **Male**: *RR*: 0.53 ± 0.03 g, *RQ*: 0.54 ± 0.03 g, *QQ*: 0.53 ± 0.02 g). IB: immunobloting. Statistical analyses are compared to respective control with Mann-Whitney U test with Bonferroni correction. n.s. (not significant), $P > 0.05$.

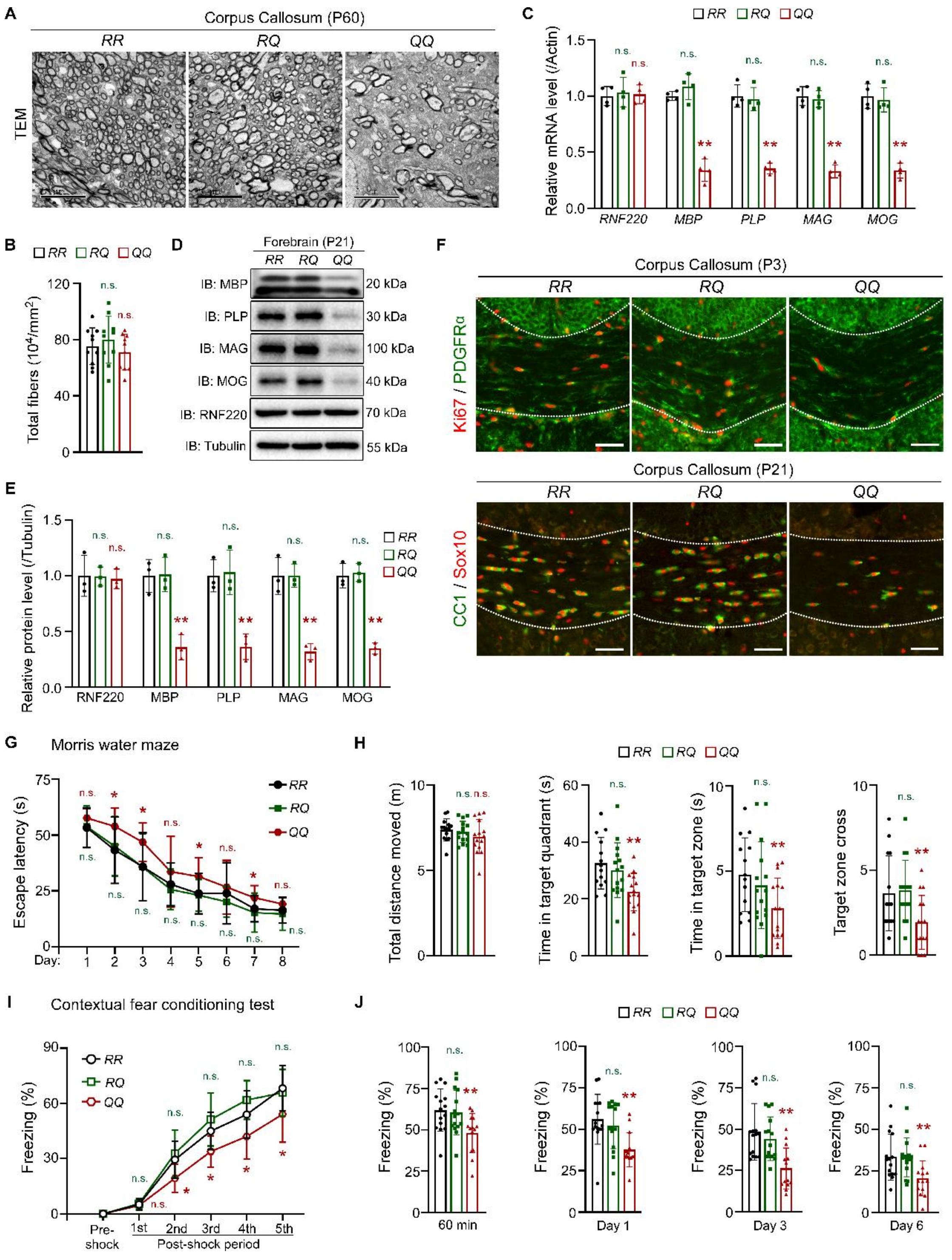

Supplementary Figure 12, related to Figure 7 and 8. OL developmental defects, hypomyelination, and impaired behaviors in RNF220^{R365Q} knock-in mice. (**A**) Electron microscopy images of the corpus callosum transverse sections from P60 *RNF220-RR* (n = 4), *RNF220-RQ* (n = 4), and *RNF220-QQ* (n = 4) mice. Scale bars: 5 μm. (**B**) Quantification of the total axon fibers in corpus callosum of RNF220-QQ mice. Bar graphs (mean ± SD) show quantification of the number of total axon fibers

(*RR*: 75.38 ± 13.03 × 10^4 / mm^2; *RQ*: 80.16 ± 16.81 × 10^4 / mm^2; *QQ*: 71.28 ± 12.70 × 10^4 / mm^2). (**C**) Realtime-PCR analyses of mRNA expression of *RNF220, MBP, PLP, MAG,* and *MOG* in the cerebral cortex of P21 *RNF220-RR* (n = 4), *RNF220-RQ* (n = 4), and *RNF220-QQ* (n = 4) mice, and bar graphs (mean ± SD) show the relative mRNA levels normalized against indicated genes expression in the respective wild-type controls (***RNF220***: *RR*: 1.00 ± 0.08, *RQ*: 1.03 ± 0.13, *QQ*: 1.02 ± 0.08; ***MBP***: *RR*: 1.00 ± 0.04, *RQ*: 1.08 ± 0.11, *QQ*: 0.34 ± 0.10; ***PLP***: *RR*: 1.00 ± 0.10, *RQ*: 0.97 ± 0.10, *QQ*: 0.35 ± 0.05; ***MAG***: *RR*: 1.00 ± 0.08, *RQ*: 0.97 ± 0.07, *QQ*: 0.33 ± 0.06; ***MOG***: *RR*: 1.00 ± 0.11, *RQ*: 0.97 ± 0.11, *QQ*: 0.34 ± 0.07). (**D** and **E**) Western blotting analyses of protein expression of RNF220, MBP, PLP, MAG, and MOG in the cerebral cortex of P21 *RNF220-RR* (n = 3), *RNF220-RQ* (n = 3), and *RNF220-QQ* (n = 3) mice, and bar graphs (E) (mean ± SD) show the relative levels normalized against indicated proteins expression in the respective wild-type controls (**RNF220**: *RR*: 1.00 ± 0.18, *RQ*: 0.99 ± 0.09, *QQ*, 0.97 ± 0.09; **MBP**: *RR*: 1.00 ± 0.15, *RQ*: 1.01 ± 0.15, *QQ*: 0.97 ± 0.09; **PLP**: *RR*: 1.00 ± 0.14, *RQ*: 1.03 ± 0.20, *QQ*: 0.36 ± 0.12; **MAG**: *RR*: 1.00 ± 0.17, *RQ*: 1.00 ± 0.11, *QQ*: 0.32 ± 0.07; **MOG**: *RR*: 1.00 ± 0.11, *RQ*: 1.03 ± 0.08, *QQ*: 0.35 ± 0.05). (**F**) Immunofluorescence staining assays of Ki67 and PDGFRα, or CC1 and Sox10 in the corpus callosum regions of *RNF220-WT* (n = 3), *RNF220-cHet* (n = 3) and *RNF220-cKO* (n = 3) mice on P3 or P21 as indicated. Scale bars: 50 μm. (**G** and **H**) Behavior analyses of Morris water maze tests for *RNF220-RR* (n = 15), *RNF220-RQ* (n = 15), and *RNF220-QQ* (n = 15) mice. Escape latencies (mean ± SD) to find the platform throughout the 8-day learning trials (E, **Day 1**: *RR*: 53.33 ± 8.89 s, *RQ:* 53.80 ± 9.35 s; *QQ*: 57.65 ± 4.12 s; **Day 2**: *RR*: 43.25 ± 14.90 s, *RQ*: 45.02 ± 13.30 s; *QQ*: 53.93 ± 8.19 s; **Day 3**: *RR*: 35.86 ± 15.22 s, *RQ*: 35.67 ± 14.95 s, *QQ*: 46.87 ± 8.63 s; **Day 4**: *RR*: 27.94 ± 9.57 s, *RQ*: 25.63 ± 9.12 s, *QQ*: 33.67 ± 15.83 s; **Day 5**: *RR*: 23.81 ± 9.07 s, *RQ*: 23.10 ± 7.07 s; *QQ*: 31.47 ± 8.48 s; **Day 6**: *RR*: 23.95 ± 13.71 s, *RQ*: 20.16 ± 6.90 s, *QQ*: 26.71 ± 12.00 s; **Day 7**: *RR*: 17.06 ± 5.82 s, *RQ*: 15.27 ± 8.77 s, *QQ*: 21.96 ± 5.46 s; **Day 8**: *RR*: 16.31 ± 5.81 s, *RQ*: 14.73 ± 7.25 s, *QQ*: 19.13 ± 3.07 s). (H) Spatial memory retrieval of these mice used in (G) was examined when the platform was removed. Bar graphs show the total

distance moved (*RR*: 739.10 ± 64.42 cm; *RQ*: 725.70 ± 62.49 cm; *QQ*: 699.00 ± 98.65 cm), duration in platform quadrant (*RR*: 32.58 ± 9.05 s; *RQ*: 30.09 ± 9.62 s; *QQ*: 22.42 ± 6.61 s), and in platform zone (*RR*: 4.79 ± 2.17 s; *RQ*: 4.17 ± 2.55 s; *QQ*: 2.8 ± 1.78 s), and platform zone crosses (*RR*: 3.64 ± 2.21; *RQ*: 3.80 ± 1.78; *QQ*: 1.93 ± 1.58). (**I** and **J**) Contextual fear conditioning analyses of *RNF220-RR* (n = 15), *RNF220-RQ* (n = 15) and *RNF220-QQ* (n = 15) mice by foot shock. Percentages of freezing behavior across fear conditioning sessions (G, **pre-shock**: *RR*: 0.00 ± 0.00%, *RQ*: 0.00 ± 0.00%, *QQ*: 0.00 ± 0.00%; **1st shock**: *RR*: 5.08 ± 3.01%, *RQ*: 5.53 ± 2.84%, *QQ*: 4.54 ± 1.93%; **2nd shock**: *RR*: 29.46 ± 10.11%, *RQ*: 32.80 ± 12.65%, *QQ*: 19.30 ± 7.68%; **3rd shock**: *RR*: 44.94 ± 10.12%, *RQ*: 51.28 ± 14.45%, *QQ*: 33.85 ± 8.60%; **4th shock**: *RR*: 53.96 ± 12.81%, *RQ*: 61.84 ± 10.52%, *QQ*: 42.01 ± 12.41%; **5th shock**: *RR*: 68.22 ± 12.26%, *RQ*: 65.72 ± 12.67%, *QQ*: 54.10 ± 15.26%). (J) Fear memory of these mice used in (I) was tested by exposure to the environment only. Bar graphs show the percentage of freezing behavior at 60 min (*RR*: 61.80 ± 12.79%; *RQ*: 60.46 ± 13.59%; *QQ*: 48.06 ± 11.82%), 1 day (*RR*: 55.97 ± 15.03%; *RQ*: 52.17 ± 13.88%; *QQ*: 37.60 ± 10.30%), 3 day (*RR*: 48.53 ± 17.11%; *RQ*: 44.27 ± 13.15%; *QQ*: 26.36 ± 12.28%) and 6 day (*RR*: 33.40 ± 13.84%; *RQ*: 33.21 ± 11.78%; *QQ*: 20.50 ± 10.71%) after the contextual fear conditioning. IB: immunoblotting; WT: wild-type. Statistical analyses are compared to respective control with Mann-Whitney U test with Bonferroni correction or unpaired student's *t*-test. n.s. (not significant), $P > 0.05$; *, $P < 0.05$; **, $P < 0.01$.

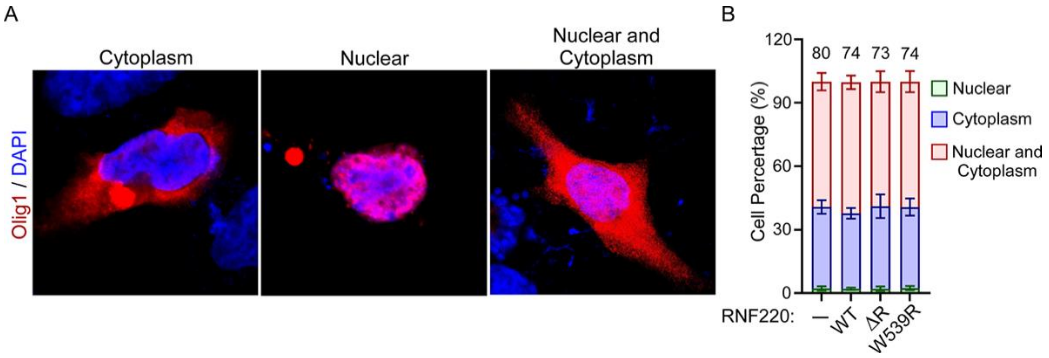

Supplemantary Figure 13, related to Discussion. The subcellular localization of Olig1 was not affected by RNF220 co-expression in HEK293 cells. (**A**) Representative images of immmunostaining assays of the subcellular localization of Olig1 when co-expressed with wild-type RNF220 or E3 ligase-dead mutants, $RNF220^{W539R}$ and $RNF220^{\Delta RING}$, in HEK293 cells. (**B**) Bar graphs (mean ± SD) show the quantification of cells with distinct subcellular localization of Olig1 (**Control**: Nuclear: 2.22 ± 0.82%, Cytoplasm: 38.57 ± 2.62%, Nuclear and Cytoplasm: 59.21 ± 3.40%; **$RNF220^{WT}$**: Nuclear: 2.02 ± 0.47%, Cytoplasm: 38.81 ± 2.05%, Nuclear and Cytoplasm: 61.84 ± 2.62%; **$RNF220^{\Delta R}$**: Nuclear: 2.11 ± 0.82%, Cytoplasm: 39.05 ± 4.55%, Nuclear and Cytoplasm: 58.84 ± 4.08%; **$RNF220^{W539R}$**: Nuclear: 2.35 ± 0.82%, Cytoplasm: 38.39 ± 3.30%, Nuclear and Cytoplasm: 59.26 ± 4.11%). WT: wild-type; ΔR: ΔRING.

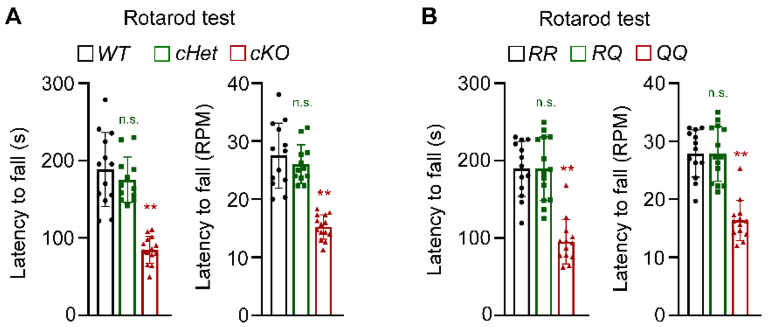

Supplementary Figure 14, related to Discussion. Performance of RNF220-cKO and RNF220^{R365Q}-KI mice in and rotarod test. (**A**) Bar graphs (mean ± SD) show the time latency to fall (*WT*: 188.40 ± 47.66 s; *cHet*: 175.00 ± 29.34 s; *cKO*: 84.90 ± 17.29 s) and the speed latency to fall (*WT*: 27.54 ± 5.58 RPM; *cHet*: 26.06 ± 3.32 RPM; *cKO*: 15.21 ± 2.09 RPM) in rotarod tests of *RNF220-cKO* mice. (**B**) Bar graphs (mean ± SD) show the time latency to fall (*RR*: 189.30 ± 35.52 s; *RQ*: 189.80 ± 41.05 s; *QQ*: 95.18 ± 28.34 s) and the speed latency to fall (*RR*: 27.90 ± 4.06 RPM; *RQ*: 27.86 ± 4.70 RPM; *QQ*: 16.38 ± 3.44 RPM) in rotarod tests of *RNF220-QQ* mice. Statistical analyses are compared to respective control with Mann-Whitney U test with Bonferroni correction. n.s. (not significant), $P > 0.05$; **, $P < 0.01$.
